# First 24-Membered Macrocyclic 1,10-Phenanthroline-2,9-Diamides—An Efficient Switch from Acidic to Alkaline Extraction of *f*-Elements

**DOI:** 10.3390/ijms241210261

**Published:** 2023-06-17

**Authors:** Pavel S. Lemport, Valentine S. Petrov, Petr I. Matveev, Uliana M. Leksina, Vitaly A. Roznyatovsky, Igor P. Gloriozov, Alexandr V. Yatsenko, Viktor A. Tafeenko, Pavel V. Dorovatovskii, Viktor N. Khrustalev, Gleb S. Budylin, Evgeny A. Shirshin, Vitaliy Yu. Markov, Alexey A. Goryunkov, Vladimir G. Petrov, Yuri A. Ustynyuk, Valentine G. Nenajdenko

**Affiliations:** 1 Department of Chemistry, Lomonosov Moscow State University, Leninskie Gory 1 bld. 3, Moscow 119991, Russia; lemport.pavel@yandex.ru (P.S.L.); vs.petrov25@gmail.com (V.S.P.); petr.i.matveev@gmail.com (P.I.M.); ulyana-air@yandex.ru (U.M.L.); vit.rozn@nmr.chem.msu.su (V.A.R.); gloriozov@nmr.chem.msu.ru (I.P.G.); yatsenko_msu@mail.ru (A.V.Y.); tafeenko-victor@yandex.ru (V.A.T.); markoff5@yandex.ru (V.Y.M.); aag@thermo.chem.msu.ru (A.A.G.); vladimir.g.petrov@gmail.com (V.G.P.); yuriustynyuk@gmail.com (Y.A.U.); 2 National Research Center “Kurchatov Institute”, Moscow 123182, Russia; paulgemini@mail.ru; 3 Department of Inorganic Chemistry, Peoples’ Friendship University of Russia (RUDN University), Moscow 115419, Russia; vnkhrustalev@gmail.com; 4 N.D. Zelinsky Institute of Organic Chemistry of Russian Academy of Sciences, Moscow 119991, Russia; 5 Laboratory of Clinical Biophotonics, Biomedical Science and Technology Park, Sechenov First Moscow State Medical University, Moscow 119991, Russia; gleb.budylin@gmail.com (G.S.B.); eshirshin@gmail.com (E.A.S.); 6 Faculty of Physics, M.V. Lomonosov Moscow State University, Moscow 119991, Russia

**Keywords:** phenanthroline, macrocycle, lanthanide, actinide, solvent extraction, DFT, NMR, XRD

## Abstract

A reaction of acyl chlorides derived from 1,10-phenanthroline-2,9-dicarboxylic acids with piperazine allows the preparation of the corresponding 24-membered macrocycles in good yield. The structural and spectral properties of these new macrocyclic ligands were thoroughly investigated, revealing promising coordination properties towards f-elements (Am, Eu). It was shown that the prepared ligands can be used for selective extraction of Am(III) from alkaline–carbonate media in presence of Eu(III) with an SF_Am/Eu_ up to 40. Their extraction efficiency is higher than calixarene-type extraction of the Am(III) and Eu(III) pair. Composition of macrocycle–metal complex with Eu(III) was investigated by luminescence and UV-vis spectroscopy. The possibility of such ligands to form complexes of L:Eu = 1:2 stoichiometry is revealed.

## 1. Introduction

Macrocyclic compounds are a hot topic in many fields of chemistry and related sciences [1,2,3,4]. In particular, macrocycles are promising ligands for applied radiochemistry and radiopharmacy [5,6]. For example, crown esters are key compounds for the selective separation of strontium-90 and caesium-137 [7,8]. Calix[n]arenes are actively being investigated as extractants for the isolation of An(III) and Cs(I) from alkaline solutions of high-level nuclear waste (HLW), which is one of the problems of nuclear heritage in Russia (Federal State Unitary Enterprise “Mayak Production Association”, Ozersk) and in the USA (Hanford, Savannah River and Oak Ridge) [9,10,11,12]. Pillar[5]arenes [13] and modified calix[4]arenes are being investigated to isolate alpha-emitting long-lived radionuclides from spent nuclear fuel (SNF) reprocessing solutions [14,15].

**Figure 1:**
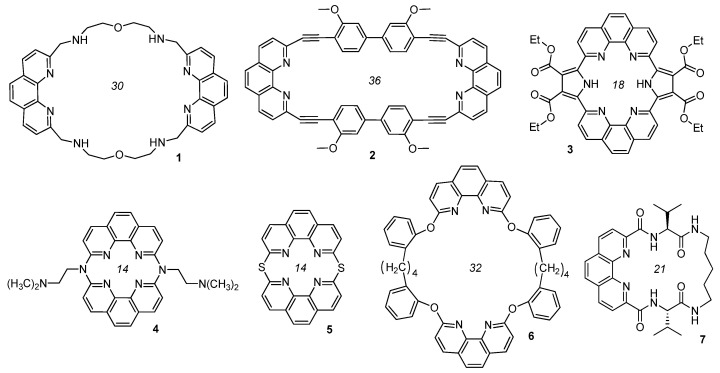
Some examples of macrocycles based on 1,10-phenanthroline core.

Strategies for the effective design of macrocyclic compounds are constantly being improved [16,17]. 1,10-Phenanthroline is one of the most commonly used nitrogen-containing building blocks for the construction of ligands for coordination and organometallic chemistry [18,19]. For example, new fluorescent chemosensors for various metal ions can be developed based on this heterocyclic core [20,21,22,23]. A number of macrocycles containing a fragment of 1,10-phenanthroline have been described. For example, ligands of the “Phen-O-Ar-” type (Figure 1) have been studied as selective complexing agents for copper ions [24,25]. 

Polyamine macrocycles “Phen-CH_2_-NH-” are known to behave as multifunctional receptors for nucleotide anions [20,21,26,27,28]. Phenanthroline macrocycles containing acetylene fragments were described, as well as mixed heterocyclic systems in which either pyrrole fragments are present or two phenanthroline rings are connected through heteroatoms [29]. Some of these macrocycles demonstrated valuable properties. For example, macrocycles combining fragments of porphyrin and 1,10-phenanthroline are an efficient fluorescent sensor for Mg(II) ions [23]. Some phenanthroline macrocycles are efficient ligands, having been used in catalytic systems [30] for azide-alkyne cycloaddition, as decarboxylation catalysts of 2-cyano-2-phenylpropanoic acid [31,32] and as DNA intercalators [33] to inhibit the enzyme that controls the continuous growth of the tumor. Some examples of phenanthroline-derived macrocycles are given in Figure 1.

So far, a wide range of compounds has been tested for the isolation of various HLW components: carbomoylphosphinoxides and diglycolamides carrying “hard” oxygen atoms as binding centers; *bis*-triazinyl-substituted heterocyclic compounds with “soft” nitrogen atoms as binding centers and various mixed N,O-donor ligands [34,35,36,37].

Diamides of 1,10-phenanthroline-2,9-dicarboxylic acid have been found highly selective extractants for the separation of actinides and lanthanides for the processing and disposal of SNF [38]. We found in the literature only one example [39] of a macrocyclic 1,10-phenanthroline-2,9-diamide **7** (Figure 1). This work is devoted to the study of the synthesis of new macrocyclic 24-membered 1,10-phenanthroline-2,9-diamides, the investigation of their structure and extraction properties toward Am(III)/Eu(III) pair. 

## 2. Results and Discussion

### 2.1. Synthesis and Structure of Macrocyclic Ligands

**Scheme 1:**
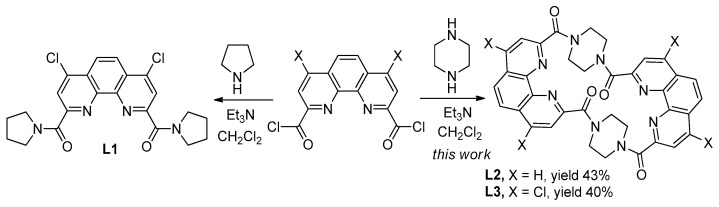
Synthesis of macrocycles **L2** and **L3**.

The extraction properties of 1,10-phenanthroline-2,9-dicarboxylic acid diamides toward f-elements can be customized by varying the structure of diamide fragments and the modification of the phenanthroline core [40,41,42,43,44,45,46,47,48]. We demonstrated earlier valuable properties of diamides prepared from cyclic amines [40,41,42]. For example, pyrrolidine-derived ligand **L1** (Scheme 1) demonstrated advanced properties in this row [41]. In this study, piperazine was chosen as the starting amine to construct two first representatives of 24-membered macrocyclic phenanthrolinediamides **L2** and **L3**. These new macrocycles were prepared in 43% and 40% yield, respectively (Scheme 1). Macrocycles **L2** and **L3** are white powders decomposing without melting at temperatures above 400 °C. They are slightly soluble in chloroform and methylene chloride, and markedly soluble in DMSO and DMF. Measuring the solubility of **L2** and **L3** in acetonitrile, chloroform and 3-nitrobenzotrifluoride (F-3), as well as evaluating their lipophilicity showed that **L3** has a noticeable solubility in F-3 and acceptable lipophilicity (Table 1) for performing the extraction tests (see Section 2.3). Therefore, most attention in this study was focused on ligand **L3**. The structures of **L2** and **L3** were studied both in the solid state (IR spectroscopy and X-ray diffraction) and in solutions (NMR spectroscopy and dynamic light scattering).

#### 2.1.1. Spectral Analysis

In the IR spectra of **L2** and **L3**, the CO-bands appear in the range from 1625 to 1640 cm^−1^ (see Figures S1 and S2 in ESI) and are split which suggests the existence of different conformers of **L2** and **L3**. 

**Figure 2:**
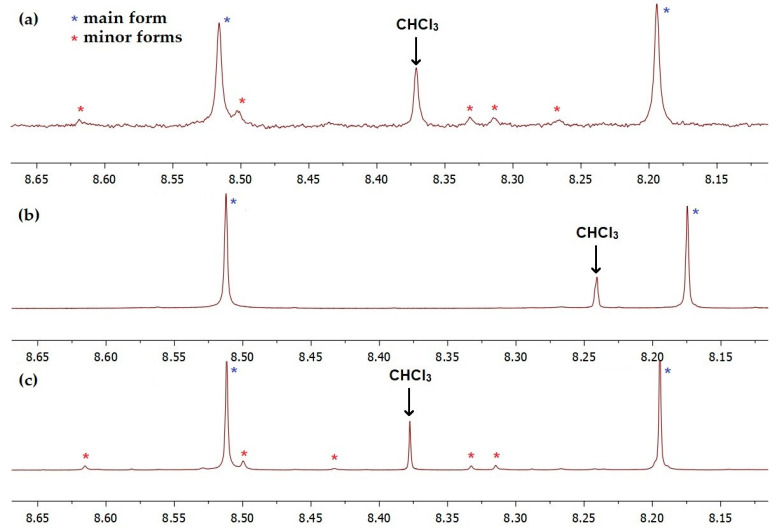
^1^H NMR (400.1 MHz) spectra of **L3** in DMF-d_7_ (**a**) after sample preparation, 25 °C; (**b**) at 70 °C; and (**c**) after cooling down to 25 °C.

The ^1^H NMR spectrum of the macrocycle **L3** in DMF at 25 °C (Figure 2), along with the signals of the main substance (marked with blue asterisks), contain additional signals of lower intensity (marked with red asterisks). With gradual heating of the sample, the broadening of all spectrum signals occurs. At 70 °C, they merge and shift towards a stronger field. The observed changes in the spectrum are completely reversible. This behavior indicates that **L3** in solution exists as an equilibrium mixture of conformers, the interconversions of which proceed fairly quickly on the NMR time scale. This behavior of **L3** and other macrocycles of this type is quite expected. Their structures contain two conformationally labile piperazine fragments, which can take chair and bath (boat) conformations, as well as four fairly conformationally labile O=C-Ar bonds. In references [40,48], we have previously observed conformational transitions at room temperature due to hindered rotation along O=C-Ar bonds in solutions of 1,10-phenanthroline-2,9-dicarboxylic acid diamides of an open structure. For more NMR spectra of macrocycles **L2** and **L3**, see ESI (Figures S3 and S4). HRMS and MALDI analyses further confirm the composition of macrocycles **L2** and **L3** (see Figures S5–S7 in ESI). 

The tendency of macrocycles **L2** and **L3** to self-aggregation will be discussed in Section 2.1.3 [49,50,51].

#### 2.1.2. Crystal Structures of **L2** and **L3** Solvates

**Figure 3:**
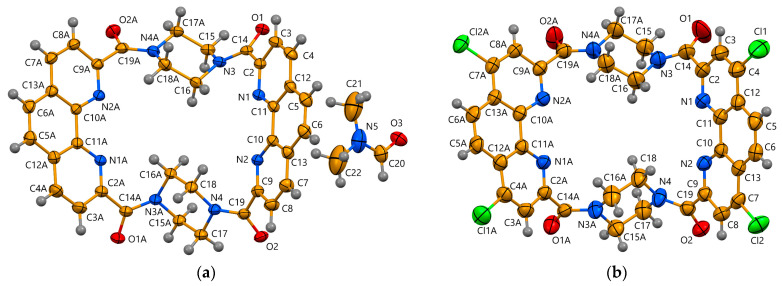
An ellipsoid (50% probability) plot of (**a**) **L2**•xDMF and (**b**) **L3**•zCHCl_3_ showing the atom labelling. Disordered DMF and CHCl_3_ molecules were removed by SQUEEZE.

**Figure 4:**
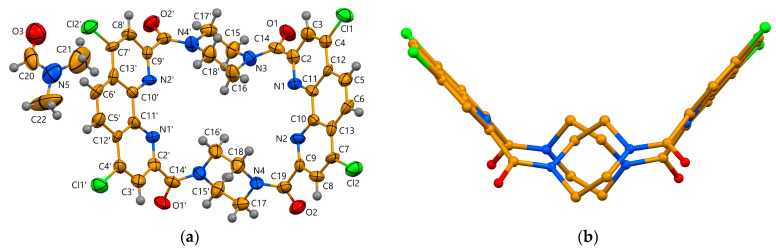
An ellipsoid (50% probability) plot of (**a**) **L3**•yDMF showing the atom labelling and (**b**) the bend of macrocyclic molecules exemplified by **L3**•yDMF. Disordered DMF molecules were removed by SQUEEZE. Atoms marked with apostrophes are related to others by the C_2_ axis (−*x*, *y*, 3/2 − *z*).

The structure of macrocycles **L2** and **L3** was unambiguously confirmed by X-ray analysis. Recrystallization of **L2** from DMF gave single crystals of **L2**· as a solvate with DMF, whereas recrystallization of **L3** from chloroform and DMF gave crystals of **L3**· solvated with CHCl_3_ and DMF, respectively. All solvate molecules were partially or completely disordered. Crystallographic characteristics of these solvates and the results of their structure determination are presented in Table S1 (see ESI). The molecular structures of the studied compounds are presented in Figure 3 and Figure 4.

In all structures, the bond dimensions are within the expected ranges [52]. In **L2**•xDMF and **L3**•yDMF, the phenanthroline fragments are essentially planar, with dihedral angles subtended by mean planes of pyridine rings being within the range from 0.5(3) to 1.46(9)°, whereas in **L3**•zCHCl_3_, one of these fragments (N1…N2) is almost planar, but another one (N1A...N2A) deviates from planarity, with dihedral angles formed by the mean planes of pyridine rings of 2.4(2) and 10.6(2)°, respectively. All macrocyclic molecules are bent, the dihedral angles formed by the mean planes of two phenanthroline fragments being 85.74(4), 90.0(1) and 84.6(1)° for **L2**•xDMF, **L3**•zCHCl_3_ and **L3**•yDMF, respectively (see Figure 4b). The amide oxygen atoms located at the greatest distance from each other form the basal plane of macrocyclic molecules.

In all three structures, the piperazine rings adopt chair conformations, but they link the phenanthroline fragments in a different manner. In **L3**•yDMF, the macrocyclic molecule lies at the crystallographic two-fold axis, in **L2**•xDMF, the molecular geometry is close to C_2_ symmetry, whereas in **L3**•zCHCl_3_, the molecular structure is close to C_s_ symmetry, with the mirror pseudoplane passing through the centers of phenanthroline fragments. The corresponding conformational transformation of macrocyclic molecule can be considered as a result of a 180-degree turn of one piperazine ring around the N···N direction. It should be noted that the **L3** molecule adopting the C_s_ conformation has the largest bending angle. 

**Figure 5:**
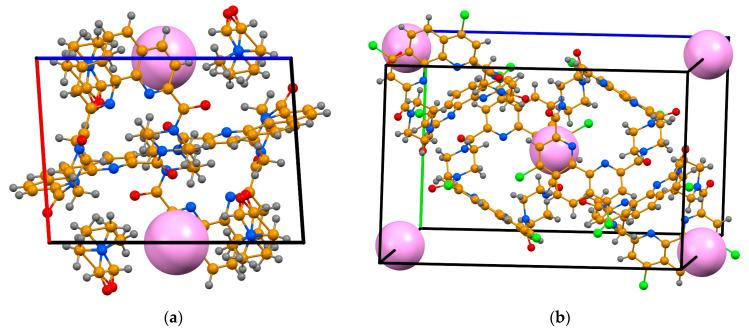
Voids in the crystal structure of (**a**) **L2**•xDMF and (**b**) **L3**•zCHCl_3_ presented by pink spheres.

The three-dimensional structures of the studied macrocyclic molecules in crystals are stabilized due to the non-classic hydrogen bonds of C-H···O type and, in some cases, by stacking interactions between the aromatic rings. In **L2**•xDMF, each molecule is linked to six neighbors by hydrogen bonds (Figure S8, Table S2). In addition, the inversion-related molecules (symmetry operations 2 − x, 2 − y, 1 − z and 1 − x, 1−y, 2 − z) are joined by stacking interactions into chains along the [1 1 −1] direction (Figure S9). In **L3**•zCHCl_3_, the molecules are joined by C-H···O contacts into chains along the *b*-axis direction (Figure S10 and Table S2). Significant stacking interactions are absent from this structure. In **L3**•yDMF, the molecules of **L3** are connected by C-H···O contacts into layers parallel to (0 1 0) (Figure S11). Furthermore, stacking interactions join the pairs of inversion-related molecules (symmetry operation −x, 1 − y, 1 − z) into dimers (Figure S12). The macrocyclic molecules are not tightly packed in the crystals, and all structures contain voids filled by solvent molecules. In the triclinic (Z = 2) crystal structure of **L2**•xDMF, there is only one void of 478 Å^3^ per unit cell centered at 0.0, 0.5, 0.5. In the monoclinic (Z = 4) crystal structure of **L3**•zCHCl_3_, there are two symmetry-related voids per unit cell, each of 565.5 Å^3^ (Figure 5b).

**Figure 6:**
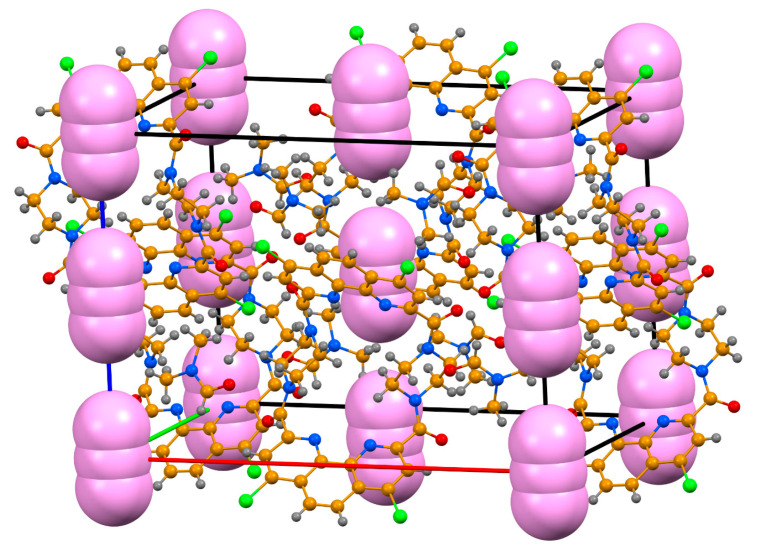
Elongated voids in the crystal structure of **L3**•yDMF presented by combinations of pink spheres.

In the orthorhombic (Z = 4) crystal structure of **L3**•yDMF, there are four symmetry-related voids per unit cell, each of 157.4 Å^3^. The voids are elongated along the b-axis direction (Figure 6).

#### 2.1.3. Dynamic Light Scattering (DLS)

Previously, the formation of aggregates has repeatedly been shown for many macrocyclic systems [49,50,51,53,54]. DLS is a commonly used method to measure hydrodynamic diameters of proteins, nanoparticles, micelles, and emulsions from several nanometers up to several micrometers [55]. This method is based on the measurement of dynamic fluctuations in light scattering intensity caused by the Brownian motion of particles. The determination of the diffusion coefficients of particles can be obtained by the analysis of the intensity fluctuations. From the diffusion coefficient measurements, one can determine the Stokes hydrodynamic radius via the Stokes–Einstein equation [56].

**Figure 7:**
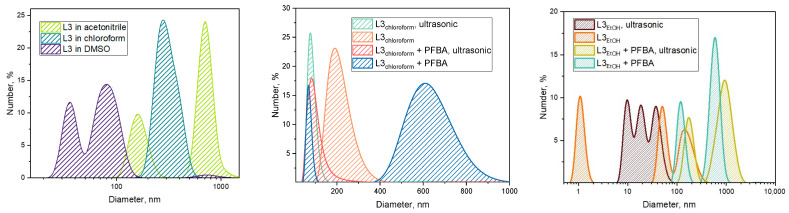
Size-distribution of **L3** in solutions: left—**L3** particles size in acetonitrile, chloroform and DMSO; center—**L3** recrystallized from chloroform in F-3 diluent; right—**L3** recrystallized from ethanol in F-3 diluent. PFBA = pentafluorobenzoic acid.

The behavior of macrocycle **L3** in solution was investigated in the different solvents: F-3, chloroform, acetonitrile, DMSO. In F-3, the size of particles was studied more thoroughly to realize what form of the particles takes part in the solvent extraction process (see Section 2.3). The distribution diagrams were obtained such as average results from three scans of one solution. The ultrasonic treatment of the organic phases based on different solvents leads to different results. We investigated the size distribution of **L3** solutions via DLS with all of the solvents used in our work. As can be seen from Figure 7, in all cases, the macrocycle forms clusters (**L3**)_n_ where the value of n can vary very widely. 

Apparently, the macrocycle in a solution has been performed with molecules’ aggregates with a median size of 80 nm, while a single **L3** molecule has a diameter of about 2 nm, according to XRD studies. It was found that the way the macrocycle was isolated affects the clusters size. Depending on the solvents used (e.g., ethanol, chloroform) the size and number of types of aggregates differed. The size of aggregates was decreased after ultrasonic enforcing. It should be noted that ethanol media increase the polydispersity of the samples. In this case, the DLS analysis becomes more complicated and decreases in accuracy. The presence of pentafluorobenzoic acid (PFBA) strongly affects the aggregation and size distribution of the (**L3**)_n_ particles and should be considered in cases when PFBA is used for solvent extraction as phase compatibilizer. In our extraction experiments (see Section 2.3), the average size of **L3**-aggregated particles was in the range of 400 to 800 nm (Figure 7). Thus, using the DLS method, we have shown that the studied macrocycle **L3** is subject to self-assembly. In addition to the ultrasonic effect, the size of the associates is strongly influenced by both the type of solvent and the presence of PFBA in the system. The DLS cumulative fits the age given in Figures S13–S25 in ESI.

### 2.2. Complexation of **L3** with Eu(III) Trinitrate in Acetonitrile Solutions

To study the coordination properties of new macrocyclic ligand **L3**, its complexation with Eu(III) trinitrate in acetonitrile was investigated by luminescence and spectrophotometric titration methods. **L3** contains two coordination cavities; so, theoretically, it can bind two metal cations. As a consequence, the experimental data were processed taking into account the formation of metal/ligand stoichiometries of 1:1 and 2:1. The investigation of Eu(III) fluorescence in the system “acetonitrile—**L3**—europium nitrate” was carried out under excitation at 300 nm. At this wavelength, the Eu(III) nitrate solution in acetonitrile is practically not excited, and the excitation of the complexes is due to the transfer of excitation energy from the ligand to the europium cation. Thus, the fluorescence bands in the range 570–720 nm (ESI, Figure S26) correspond exclusively to Eu(III)-ligand complexes.

The observed peaks in the fluorescence spectrum correspond to the following transitions: 695 nm—^5^D_0_ → ^7^F_4_, 650 nm—^5^D_0_ → ^7^F_3_, 616 nm—^5^D_0_ → ^7^F_2_, 592 nm—^5^D_0_ → ^7^F_1_ and 579 nm (^5^D_0_ → ^7^F_0_) [57]. It is known [58] that by changing the type and number of ligands in europium (III) complexes in aqueous solutions, including in titration, the line corresponding to the ^5^D_0_ → ^7^F_4_ transition at 695 nm can change shape [59].

**Figure 8:**
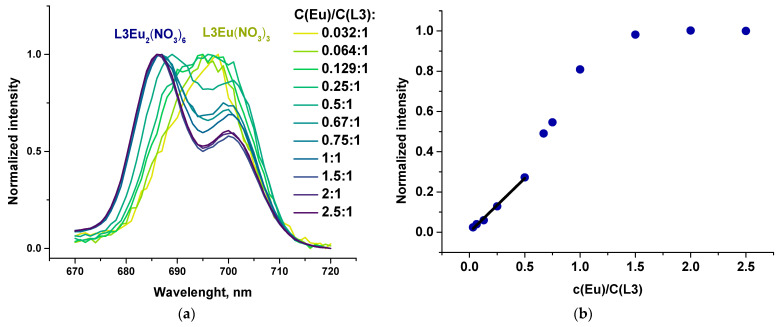
Changes in the stoichiometry of the europium complexes observed on the fluorescence spectra. (**a**) Normalized fluorescence spectrum in the region of 670–720 nm upon 300 nm excitation; (**b**) normalized dependence of line intensity in the region of 616 nm on concentration (blue circles). The solid line corresponds to a linear interpolation of the initial part of the concentration dependence.

Figure 8a shows the region of the fluorescence spectrum in the range 660–730 nm, corresponding to this transition, with normalization from zero to one in this range: I_norm_ = (I − I_min_)/(I_max_ − I_min_). As the nitrate complex in the absence of **L3** does not excite and does not luminesce, the band shape observed at the lowest concentration of europium (the yellow-green line in Figure 8a) that is at a large excess of ligand corresponds to the first stoichiometry—**L3**:Eu 1:1. As the relative concentration of europium increases, the band shape changes, indicating the formation of a new complex, which corresponds to stoichiometry **L3**:Eu 1:2 and is observed in excess of europium(III) (the dark blue line in Figure 8a). The spectra at intermediate concentrations are a weighted sum of the spectra observed at the highest and lowest concentration of europium, as there is a gradual shift in the equilibrium ratio of the concentrations of these two forms.

The dependence of the maximum intensity of the spectrum (line 616 nm) on the concentration of europium, when normalized to a value at maximum concentration, is shown in Figure 8b. The intensity dependence in Figure 8b shows that the concentration range in question can be divided into three regions: (1) initial linear growth 0:1–0.5:1; (2) growth at an increasing rate in the concentration range 0.5:1–1:1; and (3) plateau in the range 1:1-2.5:1. This dependence can be explained as follows. In the first area, there is a complex of presumably **L3**:Eu 1:1 stoichiometry; and the second region corresponds to the formation of a second complex with a higher quantum yield of luminescence. As the intensity curve in this area runs above the linear extrapolation of the intensity growth for the first complex (the dotted line in Figure 8b).

Changes in the maximum intensity corresponding to the transition ^5^D_0_ → ^7^F_2_, and changes in the shape of the strip ^5^D_0_ → ^7^F_4_ occur in sync. 

In addition to the visible changes described above, there are also some that are less pronounced. In particular, the fluorescence band maximum corresponding to the transition ^5^D_0_ → ^7^F_2_ is shifted from 616 to 618 nm. The third region corresponds to the saturated complex concentration that does not change with Eu(III) addition due to the exhaustion of ligand binding sites (Figure 8b). Thus, the dependences of the fluorescence intensity and the changes in the shape of the spectra, observed when the ratio of the europium(III) concentration to the ligand is in the range 0.03:1–2.5:1 changes, confirm the formation of two complexes, **L3**•Eu(NO_3_)_3_ and **L3**•[Eu(NO_3_)_3_]_2_. 

**Figure 9:**
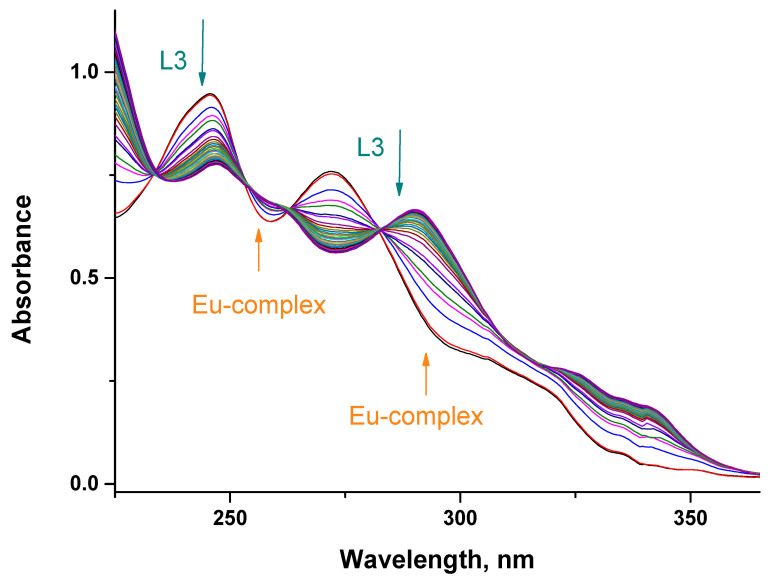
UV-Vis titration of ligand **L3** (2.5 × 10^−5^ mol/L, CH_3_CN) by Eu(NO_3_)_3_·6H_2_O (1.0 × 10^−3^ mol/L, CH_3_CN).

We performed spectrophotometric titration of **L3** with europium nitrate in acetonitrile (Figure 9).

The absorption maximum for the complex **L3**•Eu(NO_3_)_3_ occurs at 291 nm. We processed the titration data in the HypSpec2014 program and calculated the binding constants, obtaining logβ values for **L3**•Eu(NO_3_)_3_ and **L3**•[Eu(NO_3_)_3_]_2_ equal to 5.61 ± 0.05 and 8.45 ± 0.06, respectively. The values of the binding constants for the 1:1 complexes of the macrocyclic ligand **L3** and linear diamide **L1** are close [40]. A comparatively low complexation constant for the second tetradentate binding site of **L3** ($logK_{2}=log\beta_{2:1}-log\beta_{1:1}=2.84$) is probably caused by steric hindrance.

### 2.3. Solvent Extraction of Am(III) and Eu(III) 

In the initial extraction tests, it was found that the third phase is formed when the solution of **L3** in F-3 is brought in contact with water. This is probably a consequence of the amphiphilicity of the **L3** and its propensity to self-aggregate. To prevent the formation of the third phase we used organosoluble pentafluorobenzoic acid (PFBA) with a pKa of 1.48 [60]. The addition of anion-generating compounds is a common practice in the study of solvent extraction [14,61,62].

First, we conducted a blank experiment in order to establish the extracting ability of the PFBA itself. When using 0.5 mol/L PFBA solution in F-3, no extraction of Am(III) and Eu (III) was observed in either the acidic nor in the alkaline medium.

Next, we performed extraction experiments from nitric acid solutions (3 mol/L) and highly alkaline–carbonate media (pH = 11.0–13.8). Previously acyclic diamides of N-heterocyclic acids have exhibited efficient and selective extraction of Am(III) from the nitric acid. However, the distribution coefficient for the macrocycle **L3** was only D_Am_ ≈ 0.01 in nitric acid solutions. In addition, we observed a significant increase in the extraction time. In a study of complexation, we found that the binding constants to europium nitrate have close values for **L1** and **L3**. So, this decrease in the distribution ratios may be due to the lower concentration of **L3** (0.002 mol/L in solution versus 0.01–0.05 mol/L for **L1** [41]). Improving the solubility of this class of compounds will further enable this class of compounds to be used for the extraction of americium from nitric acid solutions.

**Figure 10:**
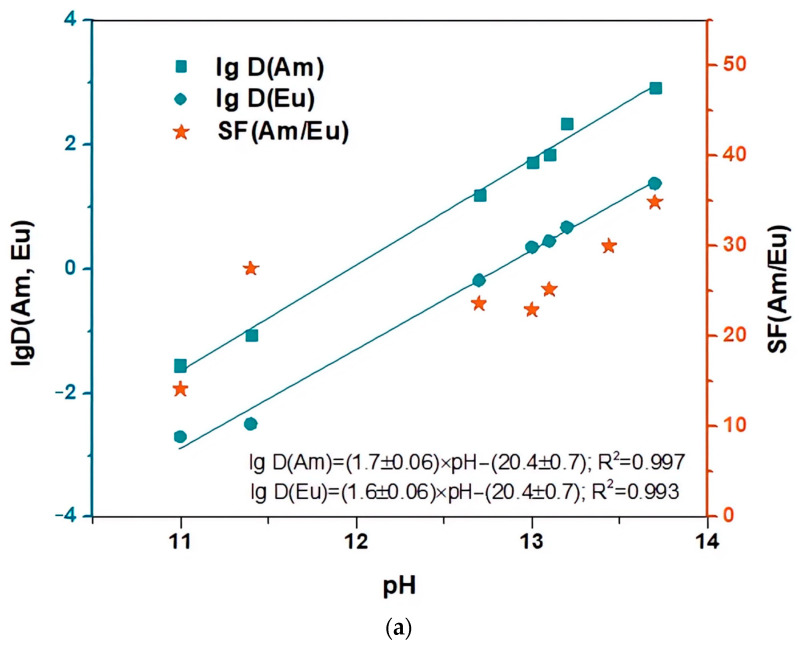

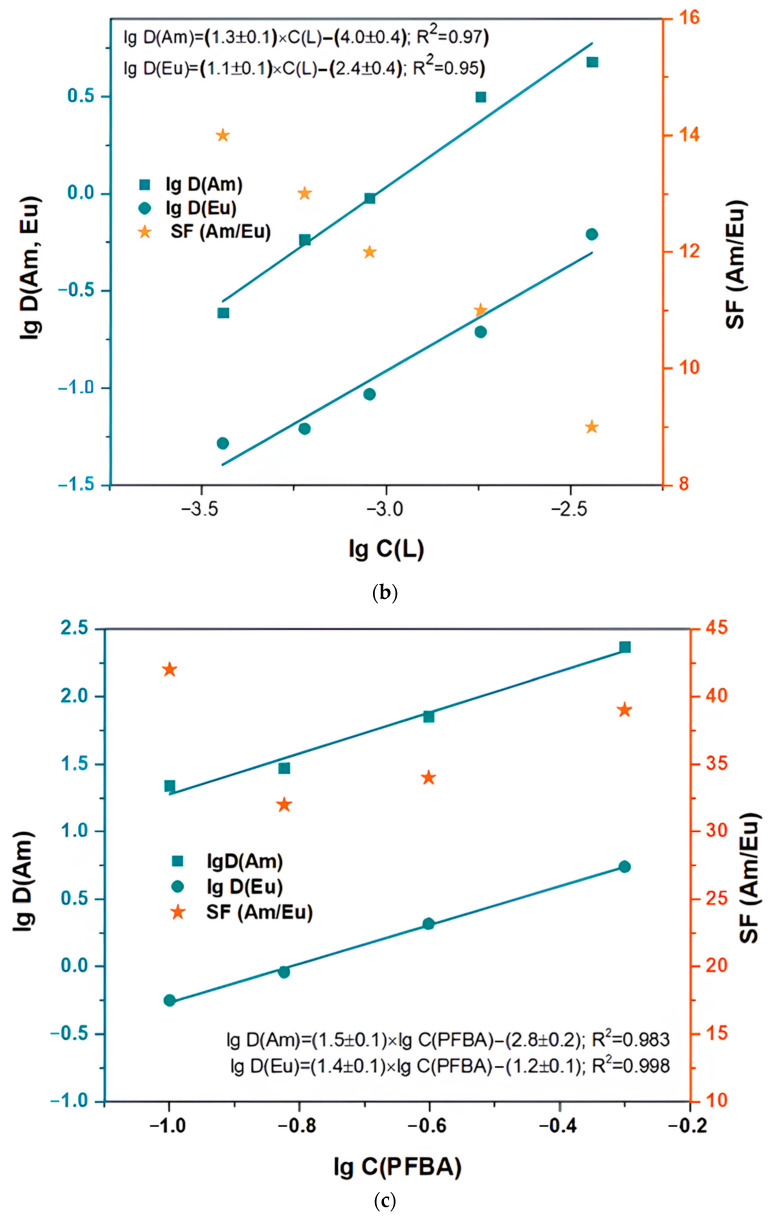
Dependence of lgD and SF_Am/Eu_ for the **L3** macrocycle-based extraction system when varying the ligand concentration, PFBA and pH value. (**a**) pH value, organic phase—2 mmol/L (**L3**), c(PFBA) = 0.5 mol/L; (**b**) lg(C(1)), pH = 13, c(PFBA) = 0.5 mol/L; (**c**) lgC(PFBA), pH = 13.1, 2 mmol/L (**L3**). Solvent—F-3, T = 25 ± 1 °C, c(Na_2_CO_3_) = 0.5 mol/L.

A practically important result was obtained in the extraction from alkaline–carbonate media. We studied the effect of pH, PFBA and ligand concentrations in solution on the extraction of Am(III) and Eu(III). The effective extraction of Am(III) was observed: the distribution ratios were in the range 1–100. It should also be noted that high SF_Am/Eu_ selectivity factors in the range of 10–40 are observed under these conditions (Figure 10). Based on the slope analysis data, one can see the formation of a 1:1 complex where both OH^−^ and PFBA act as counter-anions.

**Figure 11:**
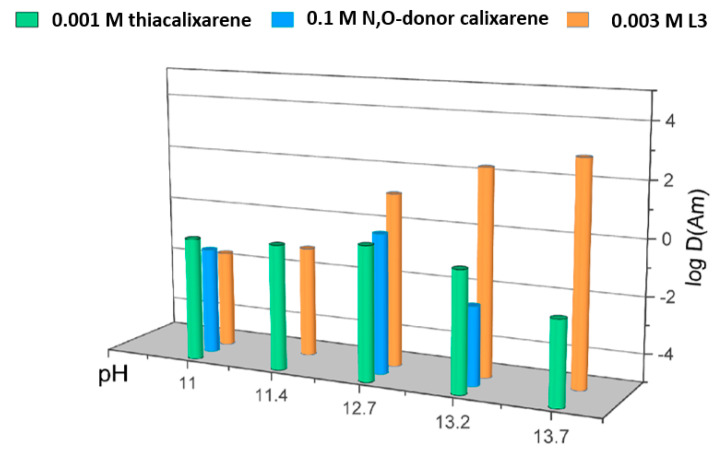
Comparison with calixarenes [63]. Diluent—F-3, T = 25 ± 1 °C.

Thus, efficient extraction is observed only when extracting from alkaline–carbonate solution and in the presence of PFBA. We compared the effectiveness of the extraction system based on macrocycle **L3** with the rare literature of examples based on calixarenes [63] under identical conditions. To our delight, the proposed new system is not inferior in its characteristics to the best representatives of this class, such as brominated tetrahydroxy-*p*-*tert*-butylthiacalix[4]arene (Figure 11). 

To elucidate the reasons for the peculiarities of the extraction behavior of **L3**, we carried out DFT modelling of the structures and formation energies of its complexes with lanthanum nitrate and hydroxonium ion (see ESI) (Equations (1) and (2)).

**L** + La(NO_3_)_3_ = **L**La(NO_3_)_3_ (1)

**L** + (H_3_O)^+^ = [**L**(H_3_O)]^+^ (2)

**Figure 12:**
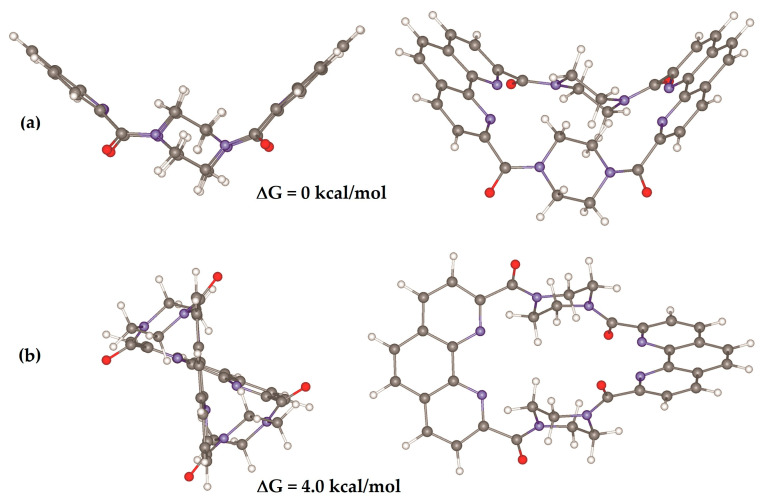
DFT-calculated structure of macrocycle **L2**: (**a**) major conformer and (**b**) a probable structure of one of its minor conformers.

The calculations showed that the most stable **L3** conformer (major conformer) has a highly symmetrical bowl structure in which both piperazine rings are in the chair conformation. This geometry (Figure 12a) is very close to that observed in the crystal (Figure 4a). Differences in bond lengths and bond angles obtained in the calculation and in the **L3** X-ray diffraction analysis do not exceed ±0.05Å and ±6°, respectively.

To bind the La^3+^ cation, the macrocycle must acquire a geometry in which there is at least one planar fragment with the *syn* orientation of both amide carbonyls, capable of acting as the N,N′,O,O′-tetradentate ligand. The potential energy surface (PES) of **L3,** as shown by our calculations, contains several local minima that meet this requirement. Among them, the most stable is the conformer whose structure is shown in Figure 12b. It is very likely that one of the minor conformers whose signals were observed in the ^1^H NMR spectrum has such a structure (Figure 2).

**Figure 13:**
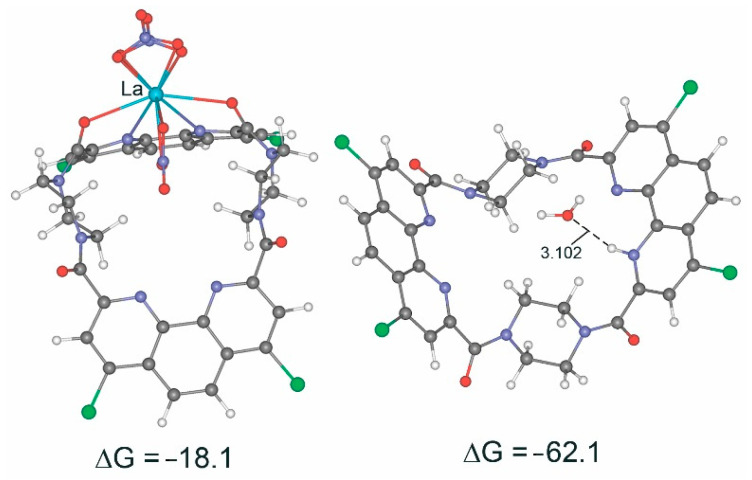
DFT-optimized structures of **L3**La(NO_3_)_3_ and **L3**[(H_3_O)^+^(NO_3_)^−^] complexes.

The calculated structures of complex **L3**La(NO_3_)_3_ and complex [**L3**(H_3_O)]^+^ are shown in Figure 13. The calculated ∆G of complex **L3**La(NO_3_)_3_ formation is −18.1 kcal/mol. This is much less than the ∆G energies for the formation of other 1,10-phenanthroline-2,9-dicarboxamides complexes with lanthanum nitrate, which vary in the range from −36 to −44 kcal/mol [40,41,42,43,44,45]. The energy of **L3** preorganization, which must be expended in order for the formation of complex **L3**La(NO_3_)_3_ to become possible, was found as high as 19.6 kcal/mol. It was calculated as a difference between the energy of a ligand in a complex and the energy of the major conformer of the ligand.

In an acid medium, the formation of a complex with a metal cation and the protonation of **L3** are two competing reactions. The formation of [**L3**(H_3_O)]^+^ proceeds without the participation of oxygen atoms of the amide groups due to only nitrogen atoms of the phenantroline core (Figure 13). It does not require considerable structural reorganization of **L3.** The formation energy is much higher (−62.1 kcal/mol) than the energy of complexation with La(NO_3_)_3_. Thus, the results of the calculations allow us to find reasons for the peculiarities of the extraction behavior of **L3**. In an acidic medium, the energy of preorganization of the rigid structure of the macrocycle **L3** hinders the formation of the complex with a metal cation. The energetic preference for the formation of the complex with the hydroxonium ion makes this direction preferable. However, the switch to an alkaline medium and the addition of hydrophobic C_6_F_5_COO^−^ anions to the system facilitates the transfer of the resulting complexes with **L3** to the organic phase to make extraction efficient.

## 3. Materials and Methods 

### 3.1. General Information

Chemical reagents such as Eu(NO_3_)_3_·6H_2_O and other inorganic/organic reagents and solvents were of analytical grade. Deuterated solvents for NMR spectra registration were purchased from commercial sources and used without further purification. 3-Nitrobenzotrifluoride (“F-3”) analytical grade was purchased from P&M Invest (Moscow, Russia) and was used as a solvent in the extraction experiments without further purification. All syntheses were performed in an argon-inert atmosphere. Dichloromethane was purified by distillation over calcium hydride prior to use. Triethylamine was purified by simple distillation, previously held for 12 h over sodium hydroxide. NMR spectra were recorded using standard 5 mm sample tubes on an Agilent 400-MR spectrometer with operating frequencies of 400.1 MHz (^1^H) and 100.6 MHz (^13^C). IR spectra in the solid state were recorded on a Nicolet iS5 FTIR spectrometer (Thermo Fisher Scientific, Waltham, MA, USA) using an internal reflectance attachment with diamond optical element−attenuated total reflection (ATR) with a 45° angle of incidence. When the resolution was 4 cm^−1^, the number of scans was 32. Positive ion MALDI mass spectra were registered using a Bruker AutoFlex II reflector time-of-flight device (N_2_ laser, 337 nm, 2.5 ns pulse). *Trans*-2-[3-(4-*tert*-butylphenyl)-2-methyl-2-propenylidene]malononitrile (DCTB, ≥98%, Aldrich, St. Louis, MO, USA) was used as a matrix, the matrix-to-analyte molar ratio in spotted probes being above 1000/1. HRMS ESI−mass spectra were recorded on the MicroTof Bruker Daltonics and Orbitrap Elite instruments. The luminescence measurements were performed on a Fluoromax-4 spectrofluorometer (Jobin Ivon). Excitation of the complexes was performed at a wavelength of 300 nm, the spectral slit width was 0.1 nm. Registration of spectra was carried out in the range of 370–720 nm, and the spectral slit registration width was 1 nm. A BS-7 filter was used to cut off the second order of scattered excitation radiation. UV-Vis absorption spectra were recorded at temperature 25.0 ± 0.1 °C in the wavelength range of 300–600 nm on a spectrophotometer (Shimadzu UV 1800) with a thermostatic attachment (Shimadzu TCC-100) using quartz cuvettes with an optical path length of 10 mm. 

A series of samples with varying europium concentration and fixed ligand concentration were prepared to evaluate the coordination changes in the europium–ligand system. The ligand concentration was chosen to be 10^−5^ mol/L and the europium concentration varied between 0.032 and 2.5 × 10^−5^ mol/L. The cuvette size (2 mm along the excitation beam) was chosen to eliminate the effect of an internal filter. 

Single crystals of **L3**•CHCl_3_ were obtained upon slow isothermal (25 °C) recrystallization of **L3** from chloroform. Single crystals **L2**•DMF and **L3**•DMF were obtained by heating the suspension of 3 mg of corresponding macrocycle in 1 mL of DMF to complete the dissolution, followed by cooling the resulting solution to room temperature. X-ray diffraction data for single crystals of **L3**•CHCl_3_ and **L3**•DMF were collected at 295 K with a Stadi Vari diffractometer (Stoe, Darmstadt, Germany) using Cu Kα radiation (=1.54186 Å). The structures were determined using SHELXT [64] and refined with SHELXL [65] programs. All non-hydrogen atoms were refined in anisotropic approximation, whereas hydrogen atoms were positioned geometrically and refined isotropically using the riding model. Absorption correction was performed using the multiscan algorithm [66]. The single-crystal X-ray diffraction data for **L2**•DMF were collected on the ‘Belok/XSA’ beamline of the Kurchatov Synchrotron Radiation Source (National Research Center ‘Kurchatov Institute’, Moscow, Russian Federation) using a Rayonix SX165 CCD detector at λ = 0.75270 Å. A total of 720 images for two different orientations of the crystal were collected using an oscillation range of 1.0° and *φ* scanning mode. The data were indexed and integrated using the utility *iMOSFLM* from the CCP4 program suite [67] and then scaled and corrected for absorption using the *Scala* program [68]. In **L2**•DMF only one DMF molecule was localized, and disorder was also observed for it in the 85:15 ratio. In **L3**•CHCl_3_, both solvate chloroform molecules were disordered. In **L3**•DMF, only two of three DMF molecules were localized. In order to account for X-ray scattering by disordered molecules, we used the SQUEEZE option of the PLATON program [69]. 

CCDC 2247968 (for **L2**•2.75DMF), 2170206 (for **L3**•2CHCl_3_) and 2247971 (for **L3**•3DMF) contain the supplementary crystallographic data for this paper. 

Dynamic light scattering (DLS) was performed at 25 °C with C(**L3**) = 3.2 × 10^−3^ mol·L^−1^ on a Malvern Instruments Zetasizer Nano-Z instrument (U.K.) for the characterization of the size of the particles in the solution. The 4 mW He-Ne 633 nm laser was used to illuminate the sample, the intensity of light scattered at an angle of 173° was measured by the avalanche photodiode. Hydrodynamic diameters of the particles were estimated from the auto-correlation function, using the Cumulants method. The size distribution curves were obtained through a Non-Negative Least Square (NNLS) method [70] The holding time of the samples was similar to the preparation conditions for the extraction experiment—1 h at room temperature. The experiment was conducted three times for each system.

### 3.2. Synthesis of the Macrocycles

In a 1 L flask in the argon atmosphere, a dry CH_2_Cl_2_ (200 mL) solution of piperazine (2 mmol, 172.3 mg) and triethylamine (5 mmol, 0.7 mL) in CH_2_Cl_2_ (200 mL) and a solution of corresponding acyldichloride (2 mmol) in 200 mL of dry CH_2_Cl_2_ were simultaneously added dropwise at room temperature under stirring. Then, the reaction mixture was stirred at ambient temperature for 72 h. Next, the reaction mixture was concentrated in vacuo, washed with water (3 × 100 mL), dried over sodium sulfate, and the solvent was distilled off. The residue was purified by flash chromatography using CH_2_Cl_2_/EtOH (3/1) mixture as an eluent, yielding the desired macrocycle **L2** or **L3**.

Macrocycle **L2**


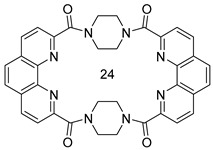


Yield 43% (274 mg), off-white solid, T._decomp._ > 400 °C. R_f_ (CH_2_Cl_2_/ethanol 3:1) = 0.2. ^1^H NMR (400 MHz, CDCl_3_) δ, ppm: 8.39 (d, *J* = 8.3 Hz, 4H), 8.09 (d, *J* = 8.3 Hz, 4H), 7.88 (s, 4H), 4.41–4.23 (m, 4H), 4.19–4.04 (m, 8H), 3.93–3.77 (m, 4H); ^1^H NMR (400 MHz, DMSO-*d*_6_) δ, ppm: 8.63 (d, *J* = 8.3 Hz, 4H), 8.08 (s, 4H), 7.96 (d, *J* = 8.3 Hz, 4H), 4.09–3.91 (m, 8H), 3.89–3.78 (m, 4H), 3.70–3.58 (m, 4H); ^13^C NMR (101 MHz, DMSO-*d*_6_) δ, ppm: 167.4 (C=O), 153.1 (Phen-C^2,9^), 143.3 (Phen-C^1′,10′^), 137.8 (Phen-C^4,7^), 128.8 (Phen-C^4′,6′^), 127.4 (Phen-C^5,6^), 123.3 (Phen-C^3,8^), 46.9 (CH_2_), 41.5 (CH_2_); IR (cm^−1^) 3046, 3013, 2916 (C-H stretching vibrations), 1633, 1622, 1617 (C=O), 1549, 1505, 1470, 1446, 1425 74 (C=C, C=N); HRMS (ESI-TOF) (*m*/*z*) [M+H]^+^ calculated for C_36_H_29_N_8_O_4_ 637.2306, found 637.2257.

Macrocycle **L3**


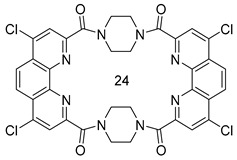


Yield 40% (310 mg), off-white solid, T._decomp._ > 400 °C. R_f_ (CH_2_Cl_2_/ethanol 3:1) = 0.4. ^1^H NMR (400 MHz, CDCl_3_) δ, ppm: 8.44 (s, 4H, Phen-CH^5,6^), 8.21 (s, 4H, Phen-CH^3,8^), 4.34–4.18 (m, 4H, CH_2_), 4.16–4.00 (m, 8H, CH_2_), 3.94–3.75 (m, 4H, CH_2_); 1H NMR (400 MHz, DMSO-*d*_6_) δ, ppm: 8.43 (s, 4H), 8.22 (s, 4H), 4.11–3.85 (m, 8H), 3.86–3.70 (m, 4H), 3.69–3.54 (m, 4H); ^13^C NMR (101 MHz, DMSO-*d*_6_) δ, ppm: 166.5 (C=O), 154.3(Phen-C^2,9^), 144.8 (Phen-C^1′,10′^), 143.4 (Phen-C^4,7^), 127.0 (Phen-C^4′,6′^), 124.6 (Phen-C^3,8^), 124.5 (Phen-C^5,6^), 55.3 (CH_2_), 47.2 (CH_2_); IR (cm^−1^) 3047, 2977, 2926 (C-H stretching vibrations), 1641, 1635 (C=O), 1573, 1533, 1463, 1447 (C=C, C=N); HRMS (ESI-TOF) (*m*/*z*) [M+NH_4_]^+^ calculated for C_36_H_28_Cl_4_N_9_O_4_ 792.0984, found 792.0979; MALDI (*m*/*z*) [M+H_2_O]^-^ calculated for C_36_H_25_Cl_4_N_8_O_5_ 791.0678, found 791.064.

### 3.3. Solvent Extraction Experiments

The experiment conditions were as follows: 1—organic phase: **L3**/PFBA/F3, aqueous phase: Am/Eu spike/pH 1 (0.1M HNO_3_); 2—organic phase: **L3**/PFBA/F3, aqueous phase: Am/Eu spike/pH 2 (0.01M HNO_3_). The extraction experiment was provided in the Eppendorf test tube, the volume of organic and aqueous phase was 500 µL. After shaking with the vortex shaker for 5 min at room temperature, the phases were separated by centrifugation at 14,000 rpm for 30 s. A total of 350 µL of both phases was taken for radionuclides determination. The extraction regularity was studied with trace amounts of ^241^Am and ^152^Eu. The amounts of the radionuclides in both phases were determined radiometrically from the γ-radiation of the corresponding radionuclide. Content of ^241^Am (E_γ_ = 59.5 keV) and ^152^Eu (E_γ_ = 121.8 keV) was determined by gamma spectrometry using a high-pure germanium detector GR 3818 (Canberra Ind.)

The sample measurement time was chosen so that the uncertainty of radiometric measurements was within 10%. The distribution ratios were calculated by the formula D = A_org_/A_aq_, where A is the specific activity of the radionuclide in the organic or aqueous phase. 

### 3.4. DFT Calculations

First-principles DFT (GGA PBE), scalar-relativistic theory [71] and a relativistic full-electron basis set of TZ quality were used in the calculations. The stationary points were identified by the analysis of Hessians. The statistical formulae for a rigid rotator and harmonic oscillator were used to calculate thermodynamic functions (Gibbs energy, *G*) at 298.15 K. The atomic charges were calculated according to Hirshfeld [72]. All calculations were performed using the PRIRODA-19 program developed by D.N. Laikov [73,74].

## 4. Conclusions

A new class of macrocyclic compounds has been obtained, the structural features of it most likely representing **L3** in the solid phase have been investigated by the X-ray diffraction method. Using the DLS method, we have shown that the studied macrocycle **L3** is subject to self-assembly. In addition to the ultrasonic effect, the size of the associates is strongly influenced by both the type of solvent and the presence of PFBA in the system. Luminescent titration of the **L3** with europium nitrate in acetonitrile was carried out. The formation of Eu**L3**(NO_3_)_3_ and Eu_2_**L3**(NO_3_)_6_ complexes was observed. By spectrophotometric titration, the complexation constants were determined: it was found that the first complexation constant coincides with the constant for the acyclic analogue **L1**. The conditions for effective Am(III) and Eu(III) extraction by the macrocycle **L3** from alkaline–carbonate media were found. The separation factor for Am(III)/Eu(III) was up to 40. The efficiency and selectivity of **L3** toward Am(III)/Eu(III) separation exceeded the efficiency and selectivity of other previously described macrocycles—calixarenes. The experimental data are in perfect agreement with results of DFT calculation, predicting that in acidic media, the most favorable process is the protonation of the macrocyclic ligand but not the formation of complex with a metal cation. This compound can be seen as a starting platform for further development of extraction systems for the extraction of *f*-elements from alkaline–carbonate solutions.

## Figures and Tables

**Table 1:** Solubility of macrocycles **L2** and **L3**.

Code	X	Solubility, mol·L^−1^			LogP **
		CH_3_CN	CHCl_3_	F-3 *	
**L2**	H	<1 × 10^−5^	~1 × 10^−4^	~7 × 10^−4^	−3.78 ± 1.65
**L3**	Cl	~1 × 10^−4^	~2 × 10^−3^	~3 × 10^−3^	−1.00 ± 1.66

* 3-Nitro-benzofluoride. ** The LogP values given in Table 1 were predicted using ACD/LogP software (ACD/Labs Release: 10.0 Product Version: 10.01).

## Data Availability

Samples of the compounds are not available from the authors.

## Supplementary Materials

The supporting information can be downloaded at https://www.mdpi.com/article/10.3390/ijms241210261/s1.
