# Supplementary material for: First 24-Membered Macrocyclic 1,10-Phenanthroline-2,9-Diamides—An Efficient Switch from Acidic to Alkaline Extraction of f-Elements
Source: Int J Mol Sci. 2023 Jun 17;24(12):10261. doi: 10.3390/ijms241210261 (PMC10299701; doi:10.3390/ijms241210261)
Supplement: Supplementary file 1 [file ijms-24-10261-s001.zip › ijms-2439169-supplementary.pdf]

Supplementary information for

First 24-membered macrocyclic 1,10-phenanthroline-2,9-diamides – an efficient switch from acidic to alkaline extraction of *f*-elements

Pavel S. Lemport,[1] Valentine S. Petrov,[1] Petr I. Matveev,[1] Uliana M. Leksina,[1] Vitaly A. Roznyatovsky,[1] Alexandr V. Yatsenko,[1] Viktor A. Tafeenko,[1] Pavel V. Dorovatovskii,[2] Viktor N. Khrustalev,[3,4] Gleb S. Budylin,[5] Evgeny A. Shirshin,[6] Vitaliy Yu. Markov,[1] Alexey A. Goryunkov,[1] Vladimir G. Petrov,[1] Yuri A. Ustynyuk,[1] Valentine G. Nenajdenko[1]*

[1] Department of Chemistry, Lomonosov Moscow State University, Leninskie Gory 1 bld. 3, Moscow 119991, Russia; lemport.pavel@yandex.ru (P.S.L.); vs.petrov25@gmail.com (V.S.P.); ulya-na-air@yandex.ru (U.M.L.); petr.i.matveev@gmail.com (P.I.M.); vit.rozn@nmr.chem.msu.su (V.A.R.); gloriozov@nmr.chem.msu.ru (I.P.G.); yatsenko_msu@mail.ru (A.V.Y.); tafeenko-victor@yandex.ru (V.A.T.); markoff5@yandex.ru (V.Y.M.); aag@thermo.chem.msu.ru (A.A.G.); vladimir.g.petrov@gmail.com (V.G.P.); yuriustynyuk@gmail.com (Y.A.U.)

[2] National Research Center "Kurchatov Institute", Moscow 123182, Russia; paulgemini@mail.ru

[3] Department of Inorganic Chemistry, Peoples' Friendship University of Russia (RUDN University), Moscow 115419, Russia; vnkhrustalev@gmail.com

[4] N.D. Zelinsky Institute of Organic Chemistry of Russian Academy of Sciences, Moscow 119991, Russia

[5] Laboratory of Clinical Biophotonics, Biomedical Science and Technology Park, Sechenov First Moscow State Medical University, Moscow 119991, Russia; gleb.budylin@gmail.com (G.S.B.); eshirshin@gmail.com (E.A.S.)

[6] Faculty of Physics, M.V. Lomonosov Moscow State University, Moscow 119991, Russia

[*] Correspondence: nenajdenko@org.chem.msu.ru

Table of content

1. Spectra drawings……………………………………………………………………….S2

2. X-ray analysis data……………………………………………………………………..S9

3. DLS cumulative fits……………………………………………………………………..S12

4. Luminescence titration data…………………………………………………………....S18

5. Computation data…………………………………………………………………………S19

1. Spectra drawings

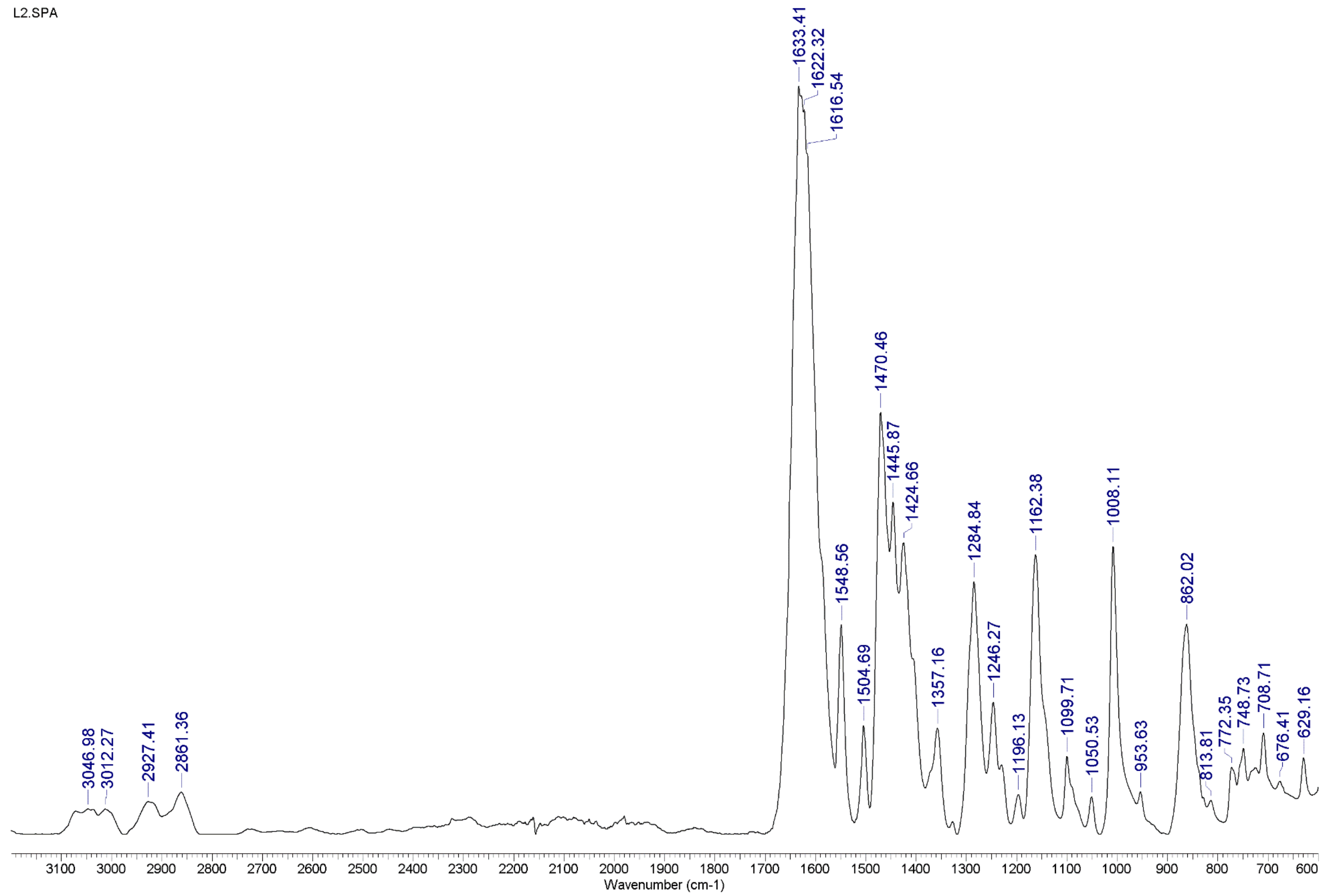

Figure S1. Solid-state IR spectrum of macrocycle **L2** at 25°C

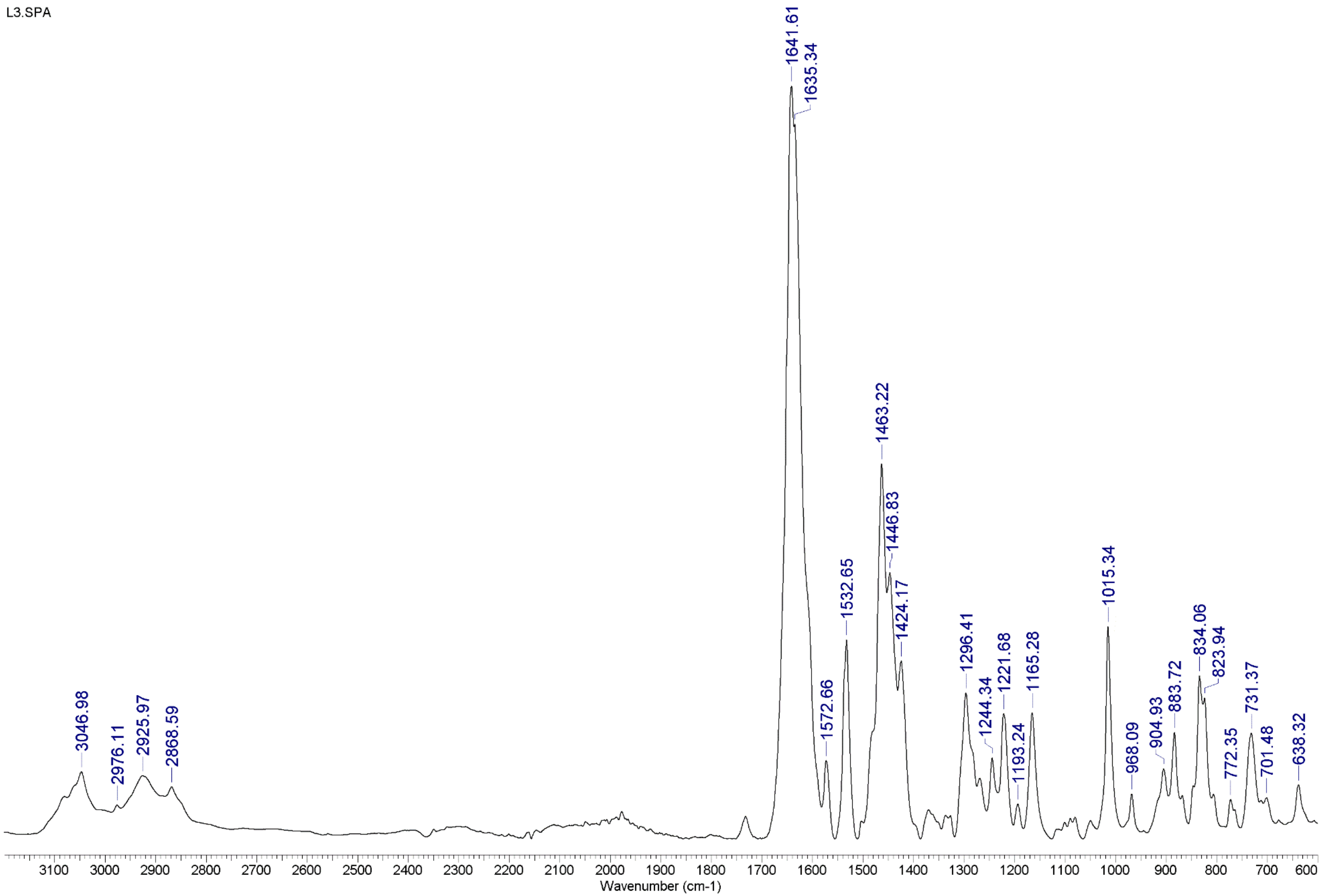

Figure S2. Solid-state IR spectrum of **L3** at 25°C

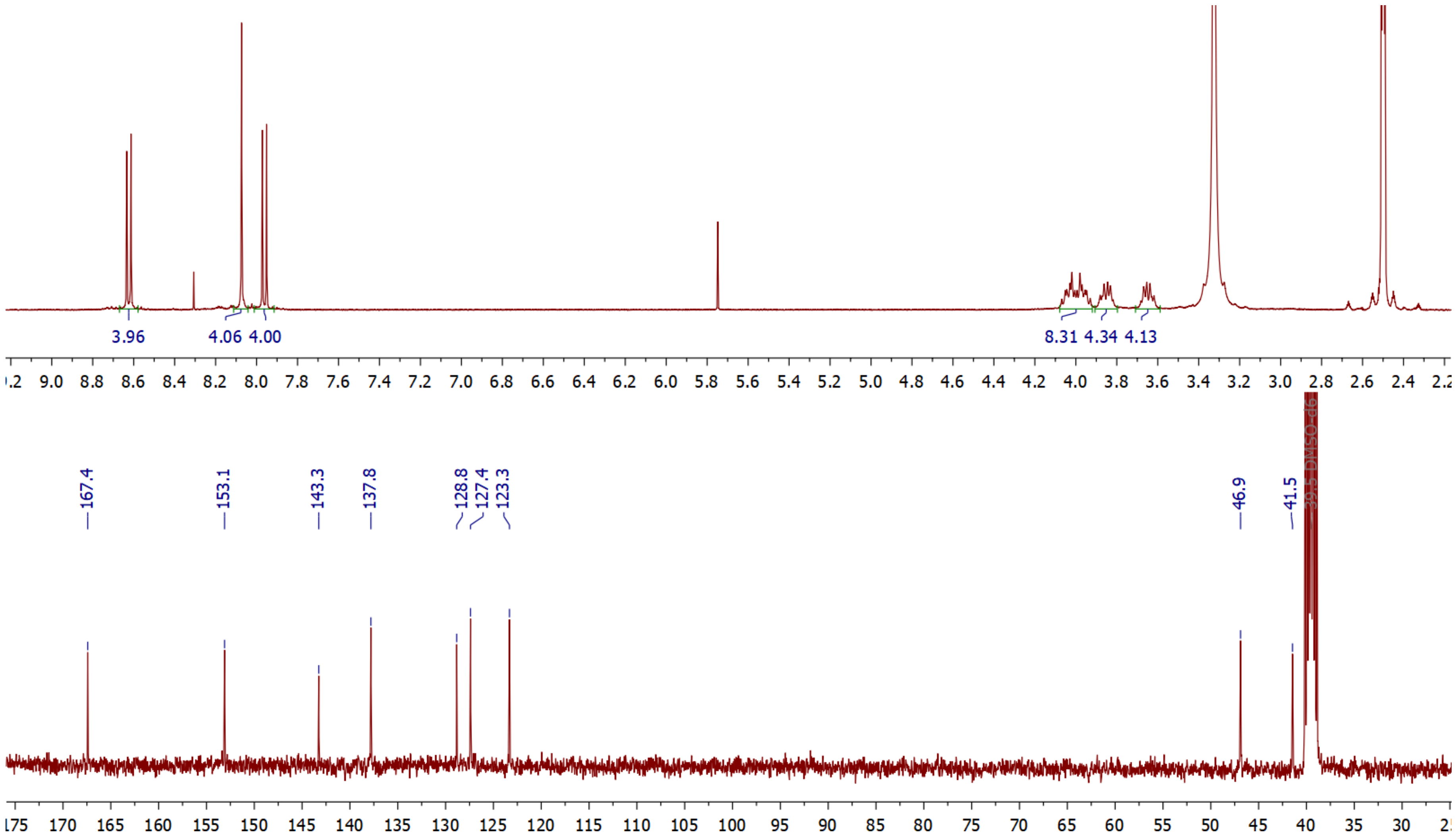

Figure S3. ^{1}H and ^{13}C NMR spectra of **L2** (DMSO-d_6, 25°C).

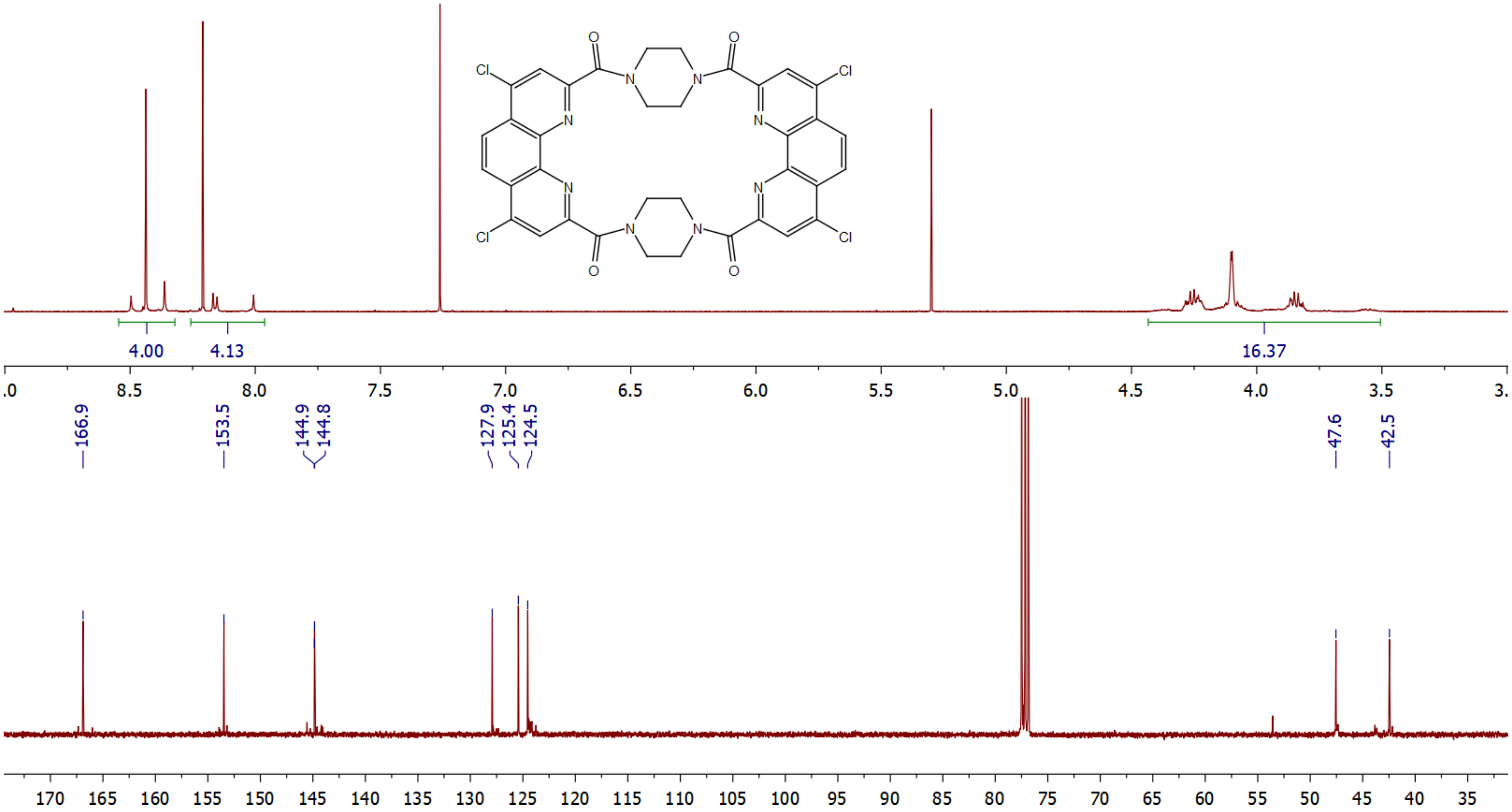

Figure S4. ^{1}H and ^{13}C NMR spectra ($CDCl_3$, 25°C)

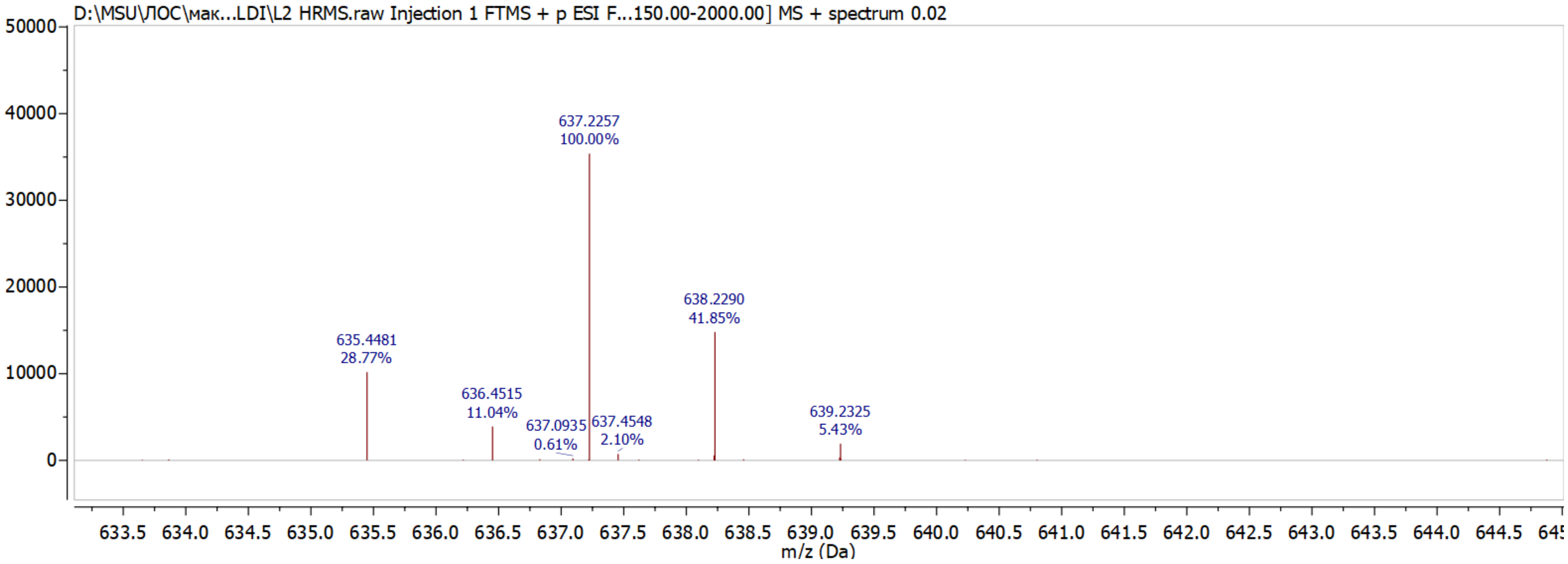

Figure S5. Fragmental view of HRMS ESI spectrum of **L2**

Display Report

Analysis Info

Analysis Name	D:\Data\Kolotyrkina\2020\Muzalevsky\0930038.d	Acquisition Date	30.09.2020 18:55:47
Method	tune_50-1600.m	Operator	BDAL@DE
Sample Name	/MUSE PVS-076	Instrument / Ser#	micrOTOF 10248
Comment	C36H24Cl4N8O4 mH 773.0747 calibrant added, CH3CN		

Acquisition Parameter

Source Type	ESI	Ion Polarity	Positive	Set Nebulizer	1.0 Bar
Focus	Not active			Set Dry Heater	200 °C
Scan Begin	50 m/z	Set Capillary	4500 V	Set Dry Gas	4.0 l/min
Scan End	1600 m/z	Set End Plate Offset	-500 V	Set Divert Valve	Waste

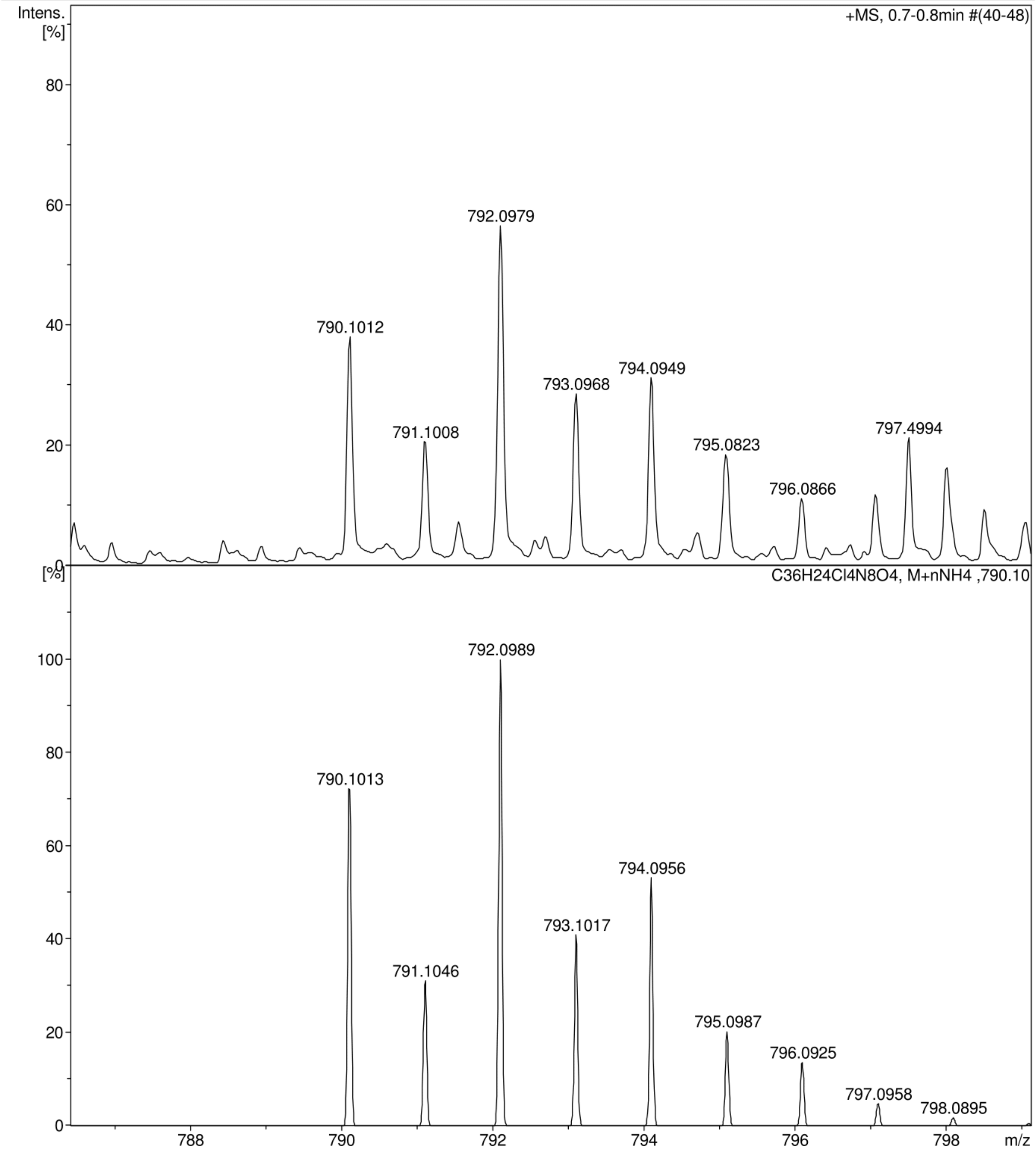

Bruker Compass DataAnalysis 4.0 printed: 30.09.2020 19:00:55 Page 1 of 1

Figure S6. Fragmental view of HRMS ESI spectrum of **L3**

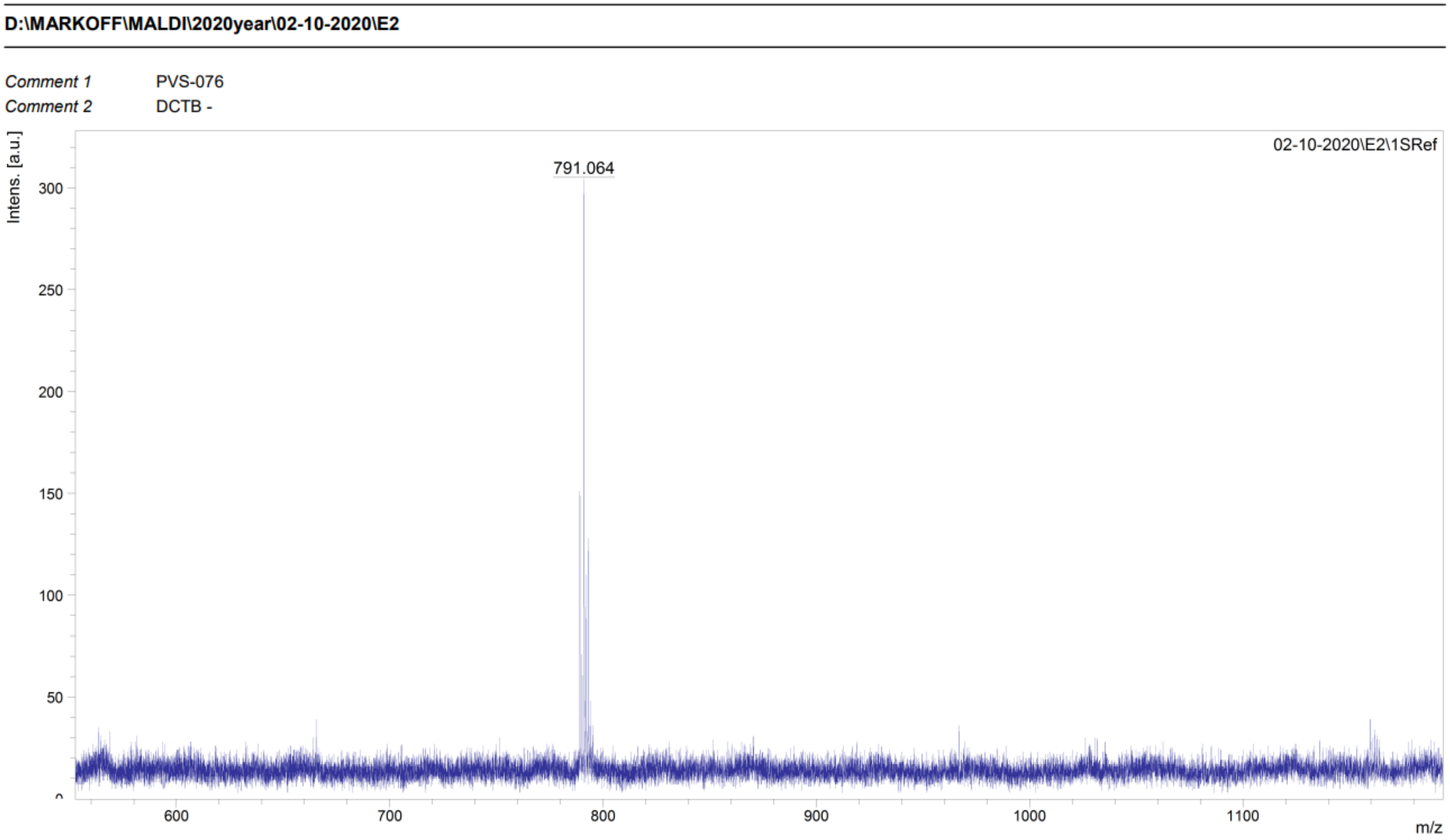

Figure S7. MALDI spectrum of macrocycle **L3**.

2. X-ray analysis data

Table S1. Crystal data and structure refinement details for **L2**·2.75DMF, **L3**·2$CHCl_3$ and **L3**·3DMF.

	**L2**·2.75DMF	**L3**·2$CHCl_3$	**L3**·3DMF
a, Å	10.9660(11)	13.3836(3)	21.7930(10)
b, Å	14.2860(14)	14.7558(4)	13.8810(10)
c, Å	15.1070(15)	22.1736(5)	15.4230(10)
α, °	69.625(10)	90.0	90.0
β, °	79.528(11)	105.844(3)	90.0
γ, °	69.554(10)	90.0	90.0
V, Å^3	2074.0(4)	4212.61(19)	4665.6(5)
Z	2	4	4
Sp. gr.	P-1	$P2_1/c$	Pbcn
λ, Å	0.75270	1.54186	0.71073
T, K	100	295	295
Θ_{max}, °	30.981	67.686	25.995
$N_{measured}$	30844	32127	58030
$N_{averaged}$/N(I>2σ)	10892 / 7534	7363 / 3592	4580 / 2064
R_{int}	0.0479	0.0813	0.1352
R (I>2σ)	0.0580	0.0476	0.0866
wR^2 (all)	0.1634	0.1262	0.1581
GoF	1.052	0.830	1.002
Δϱ, e Å^{-3}	0.596/-0.594	0.445/-0.248	0.238/-0.201
CCDC number	2247968	2170206	2247971

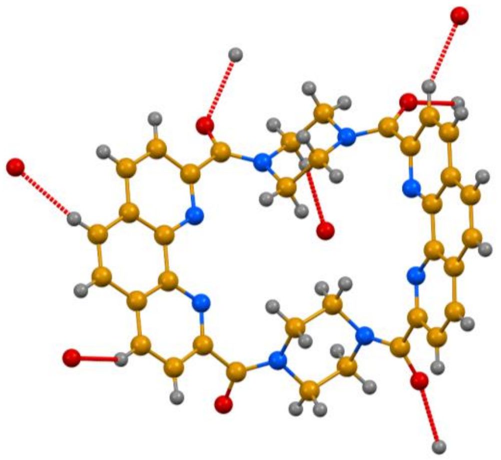

Figure S8. The C-H...O hydrogen bonds formed by **L2** molecule in **L2**·2.75DMF

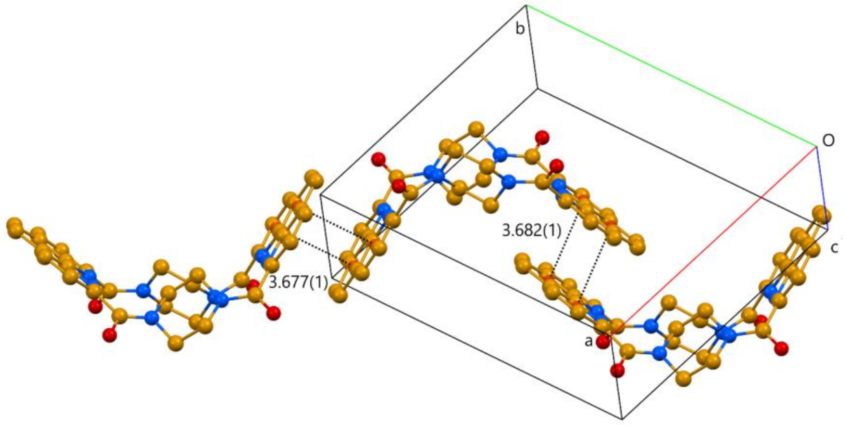

Figure S9. Stacking interactions in **L2**·2.75DMF.

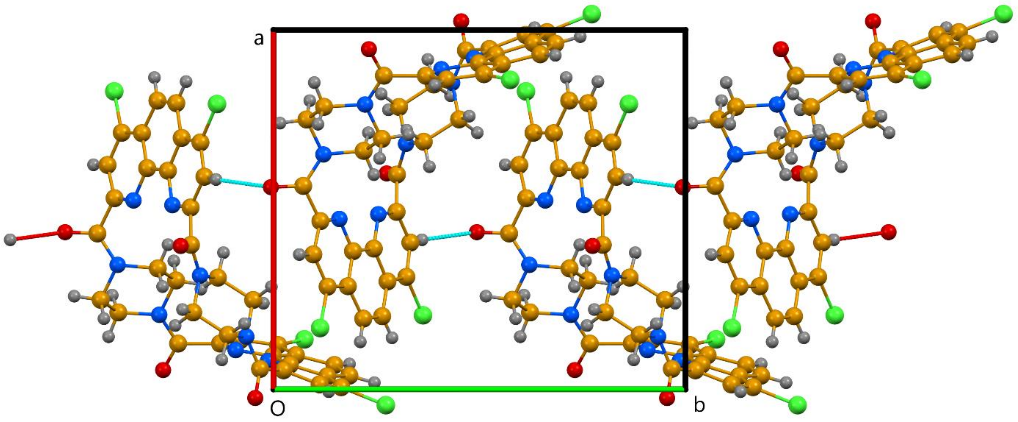

Figure S10. Chains of hydrogen-bonded **L3** molecules in **L3**·2$CHCl_3$.

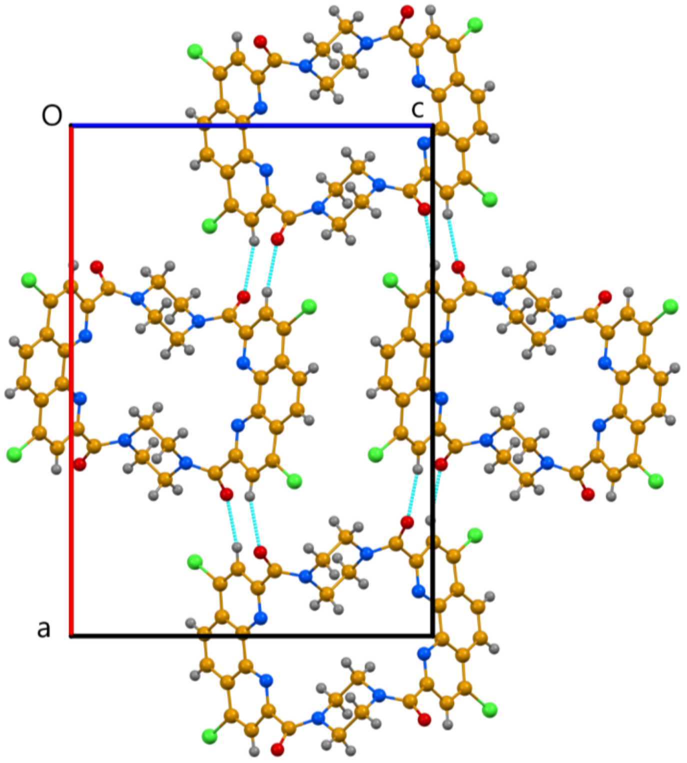

Figure S11. Layers of hydrogen-bonded **L3** molecules in **L3**·3DMF.

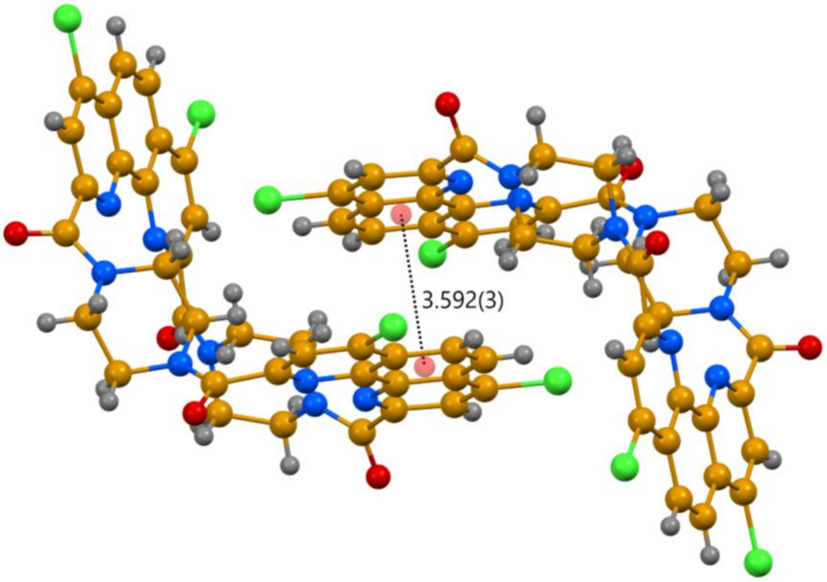

Figure S12. Stacking dimers in **L3**·3DMF.

Table S2. Non-classic C-H...O hydrogen bonds in the structures **L2**·2.75DMF, **L3**·2CHCl3 and **L3**·3DMF.

C-H···O contact, symmetry operation	C-H, Å	H···O, Å	C···O, Å	∠ C-H···O, °
**L2**·2.75DMF				
C15-H15B···O1A(1-x,2-y,1-z)	0.99	2.24	3.180(2)	159
C8A-H8A···O1(1-x,2-y,1-z)	0.95	2.41	3.281(2)	153
C7-H7···O2A(x,-1+y,z)	0.95	2.41	3.267(3)	150
**L3**·2CHCl3				
C3-H3..O2(1-x,1/2+y,3/2-z)	0.93	2.34	3.172(5)	149
**L3**·3DMF				
C3-H3..O2(-1/2+x,1/2-y,1-z)	0.93	2.29	3.204(6)	167
C8-H8..O1(1/2+x,1/2-y,1-z)	0.93	2.42	3.310(6)	162

3. DLS cumulative fits

Figure captions indicate conditions: solvent, ultrasonic treatment, presence of pentafluorobenzoic acid, recrystallisation from ethanol

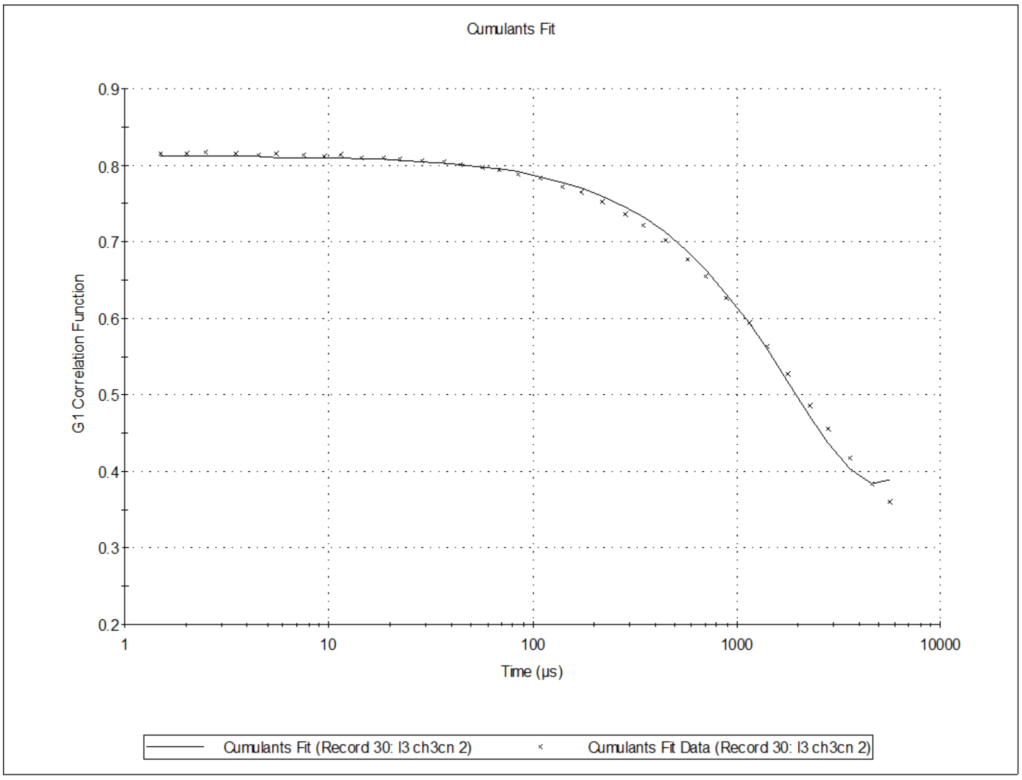

Figure S13. Acetonitrile

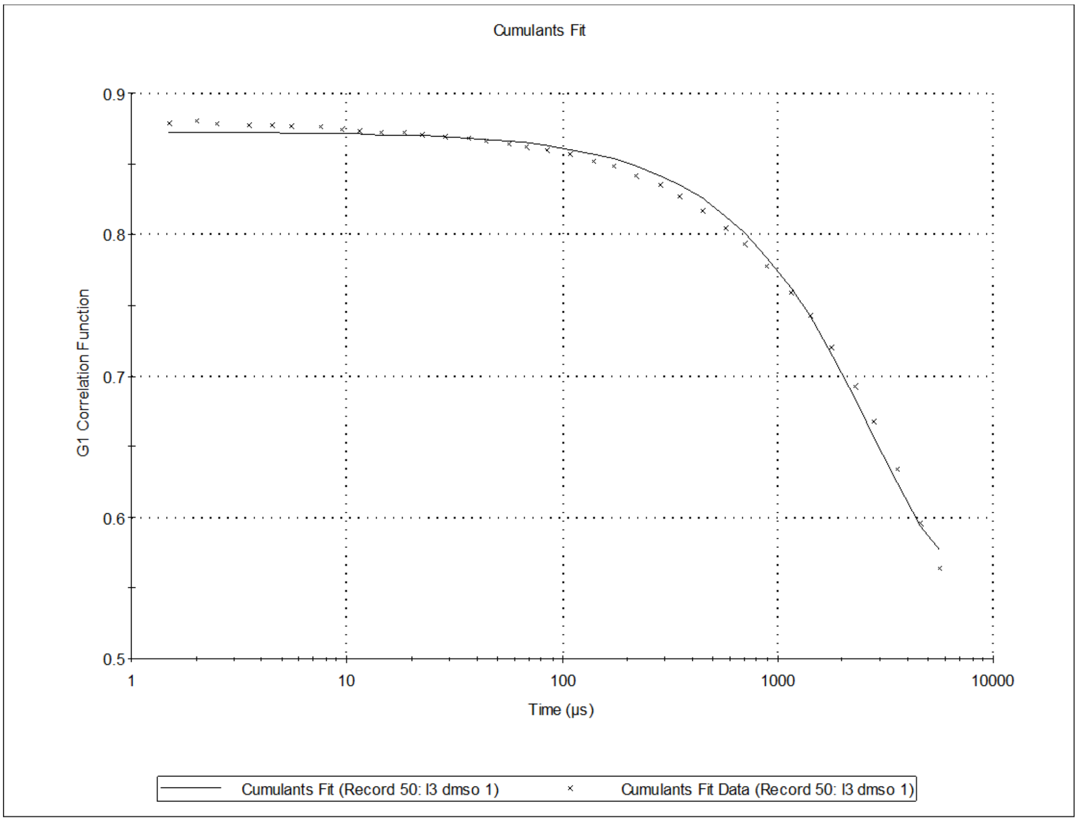

Figure S14. Chloroform

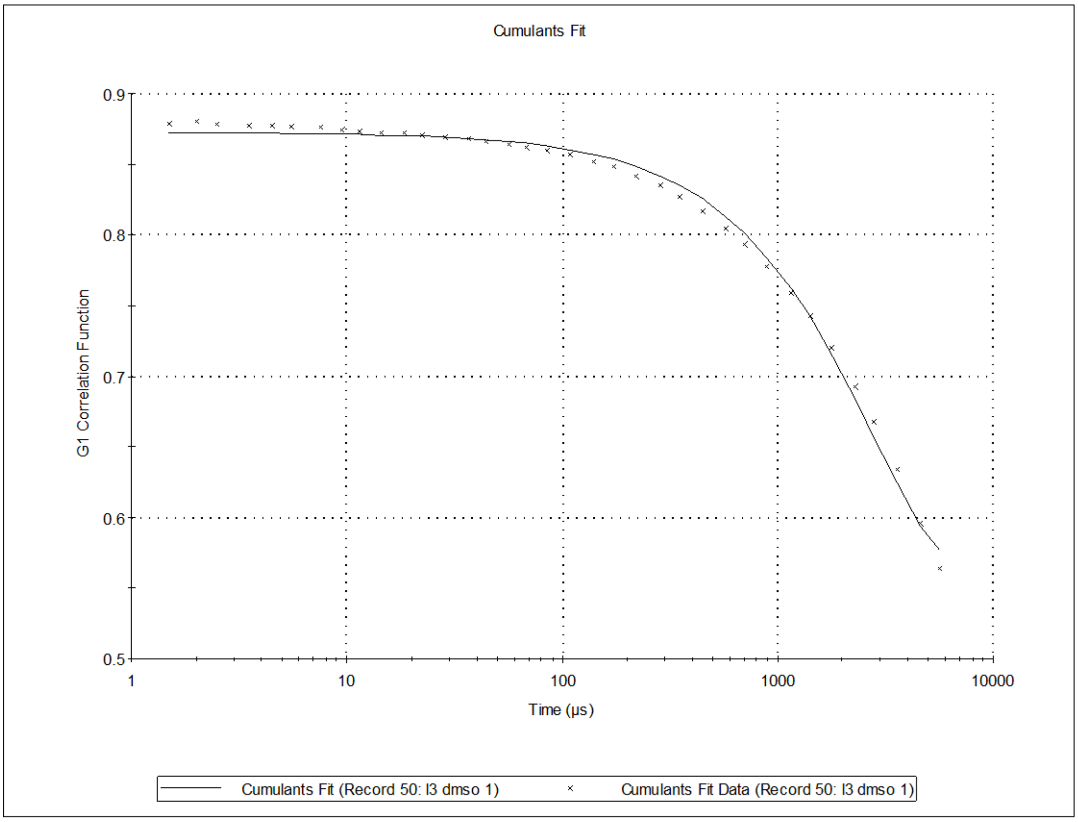

Figure S15. DMSO

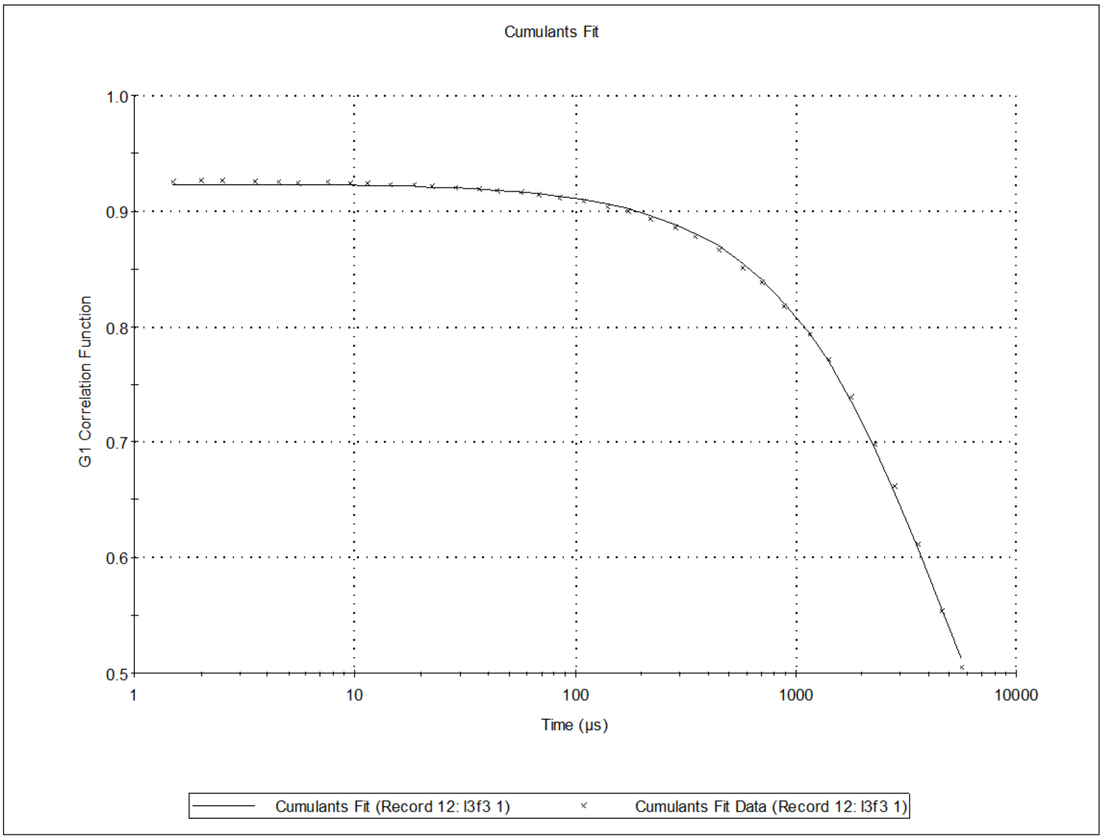

Figure S16. F-3

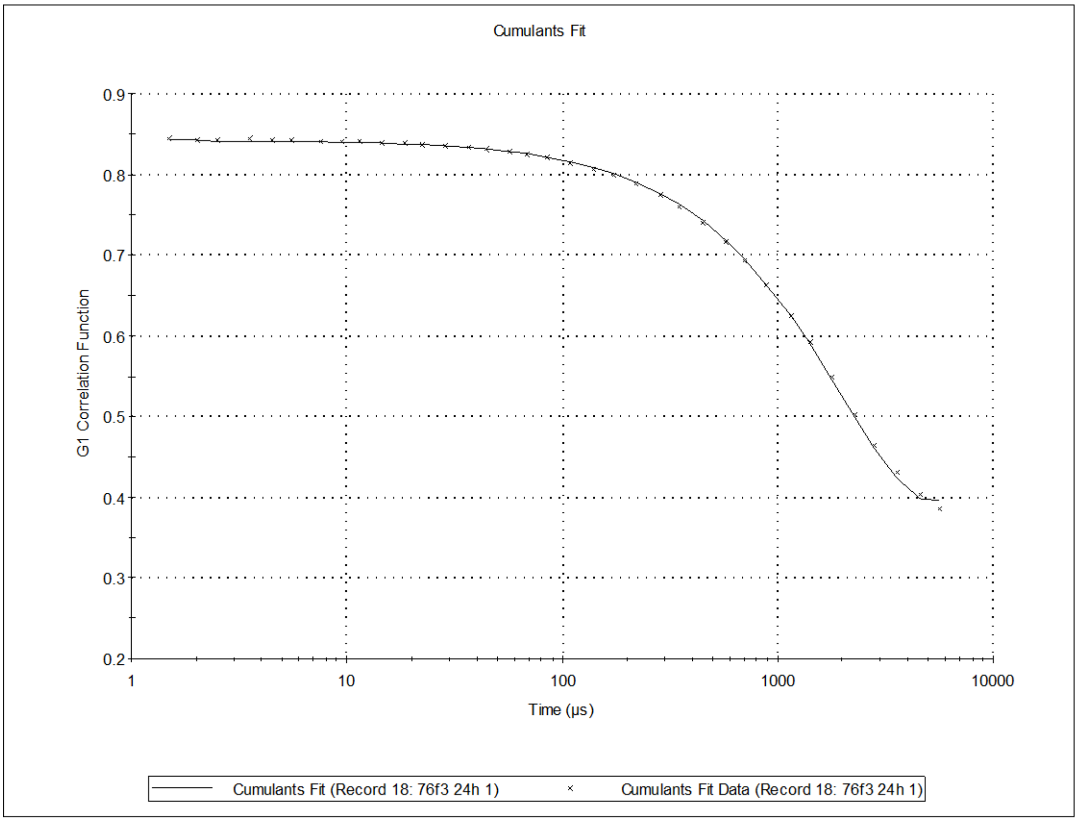

Figure S17. F-3, recrystallisation from ethanol

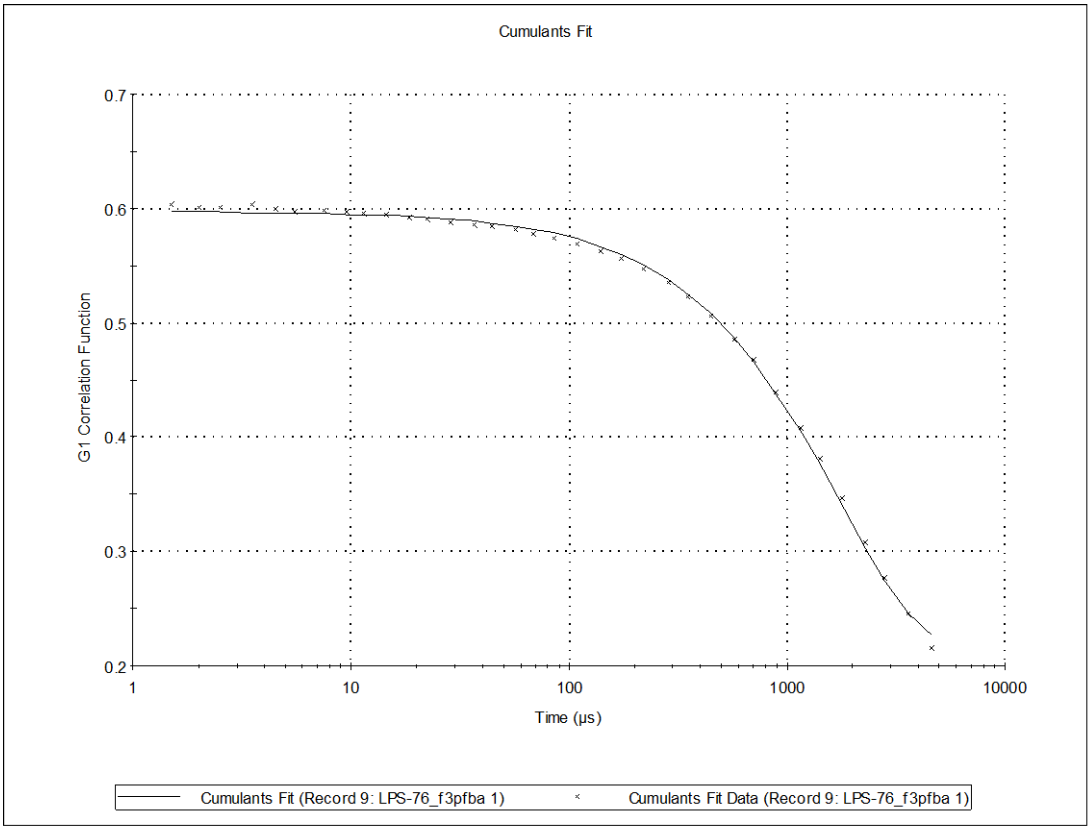

Figure S18. F-3, pentafluorobenzoic acid

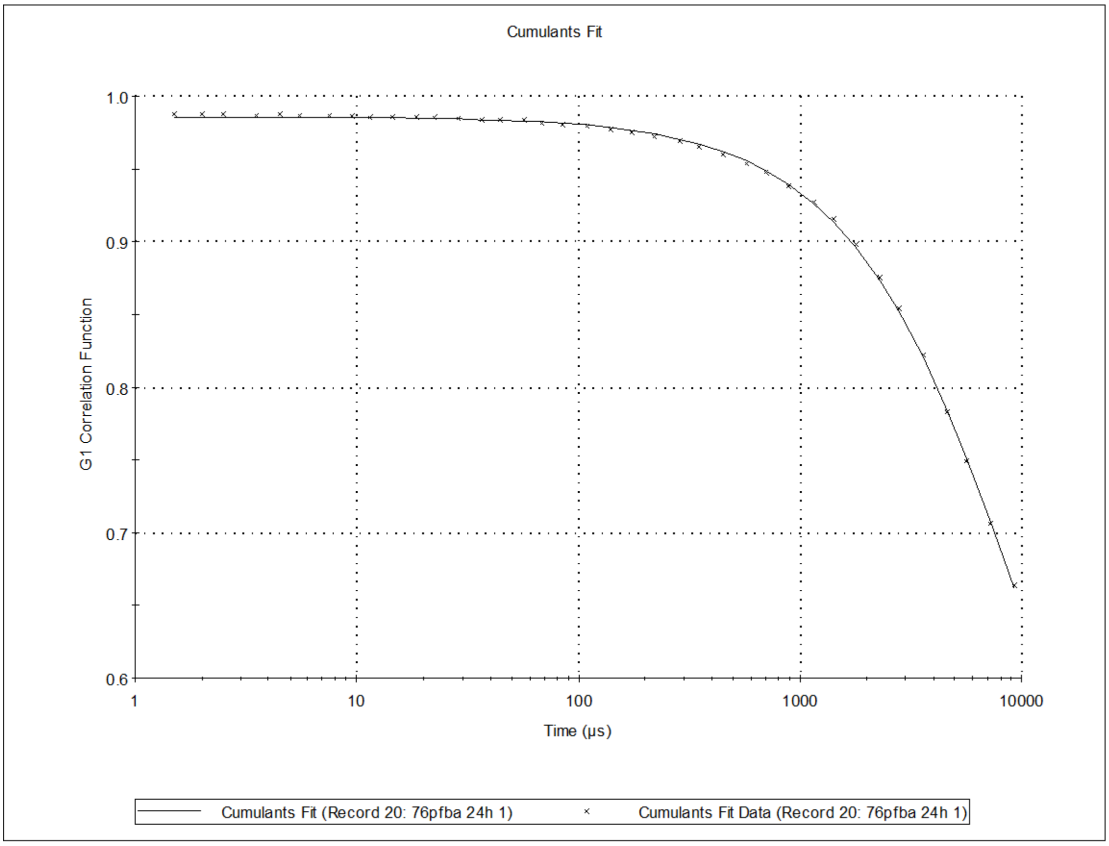

Figure S19. F-3, pentafluorobenzoic acid, recrystallisation from ethanol

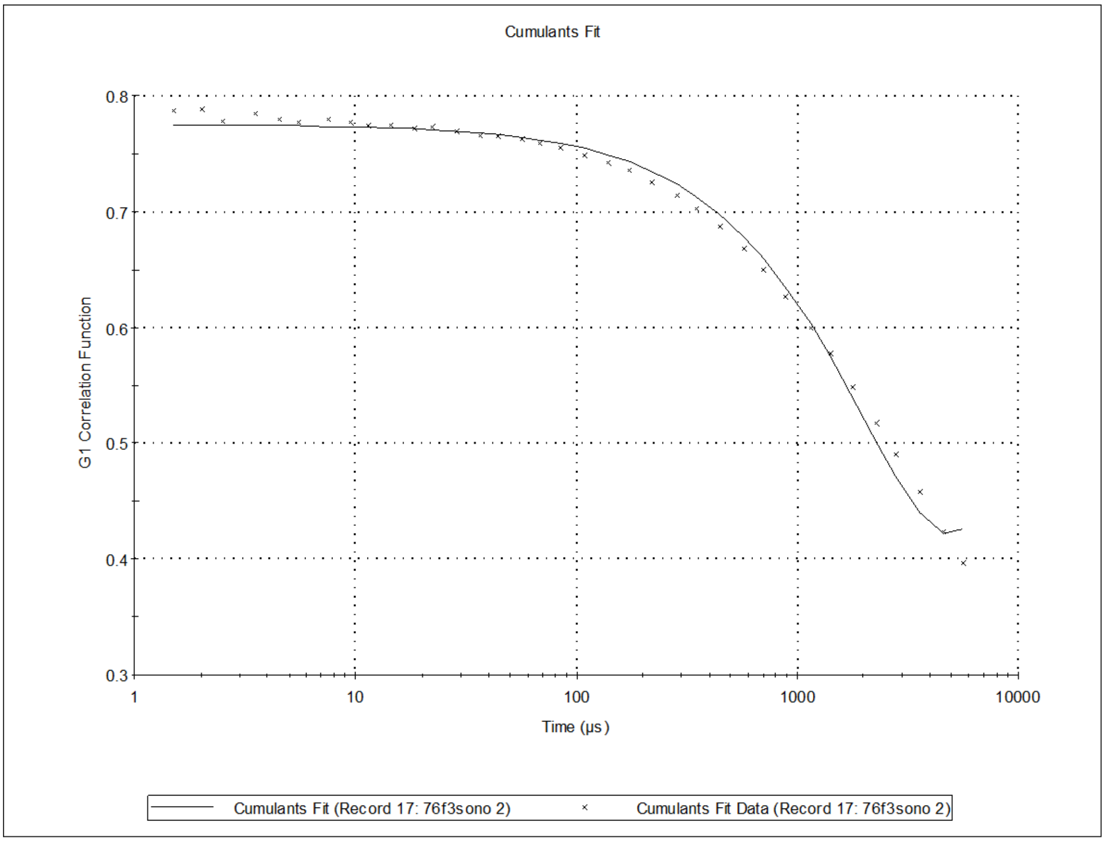

Figure S20. F-3, ultrasonic

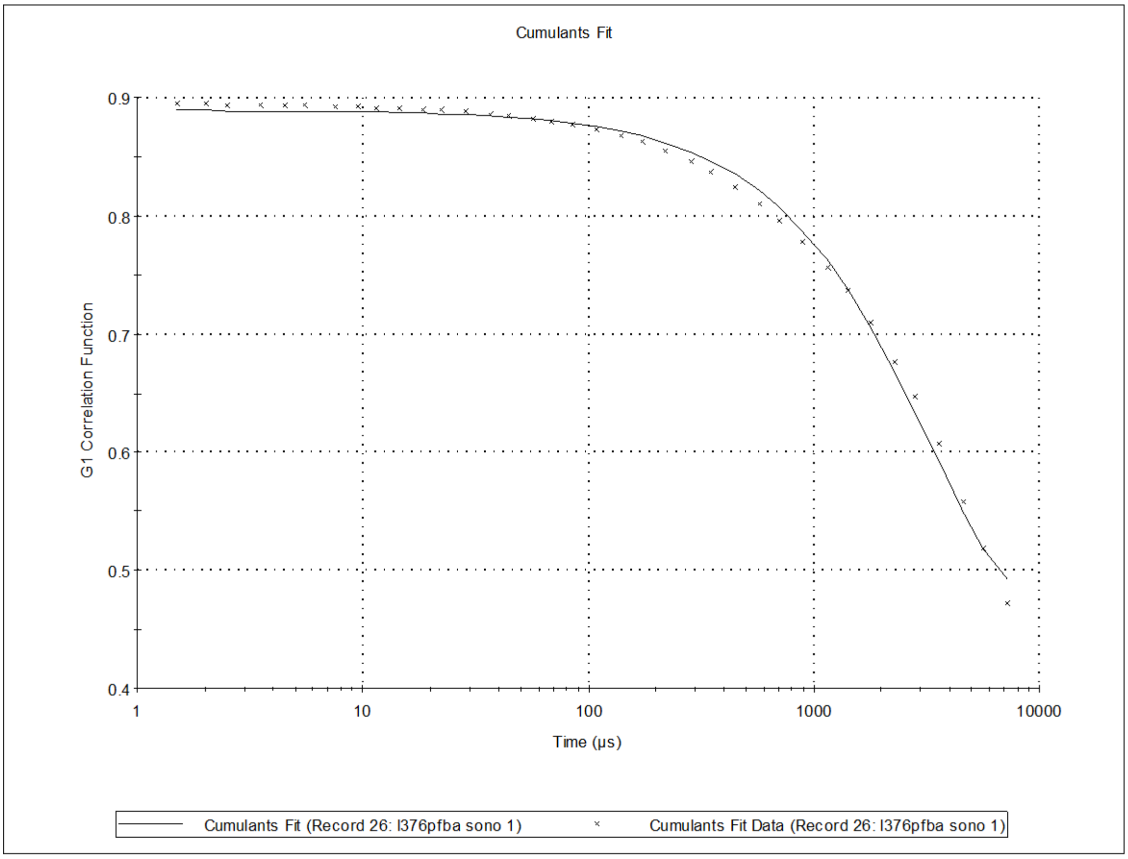

Figure S21. F-3, pentafluorobenzoic acid, ultrasonic

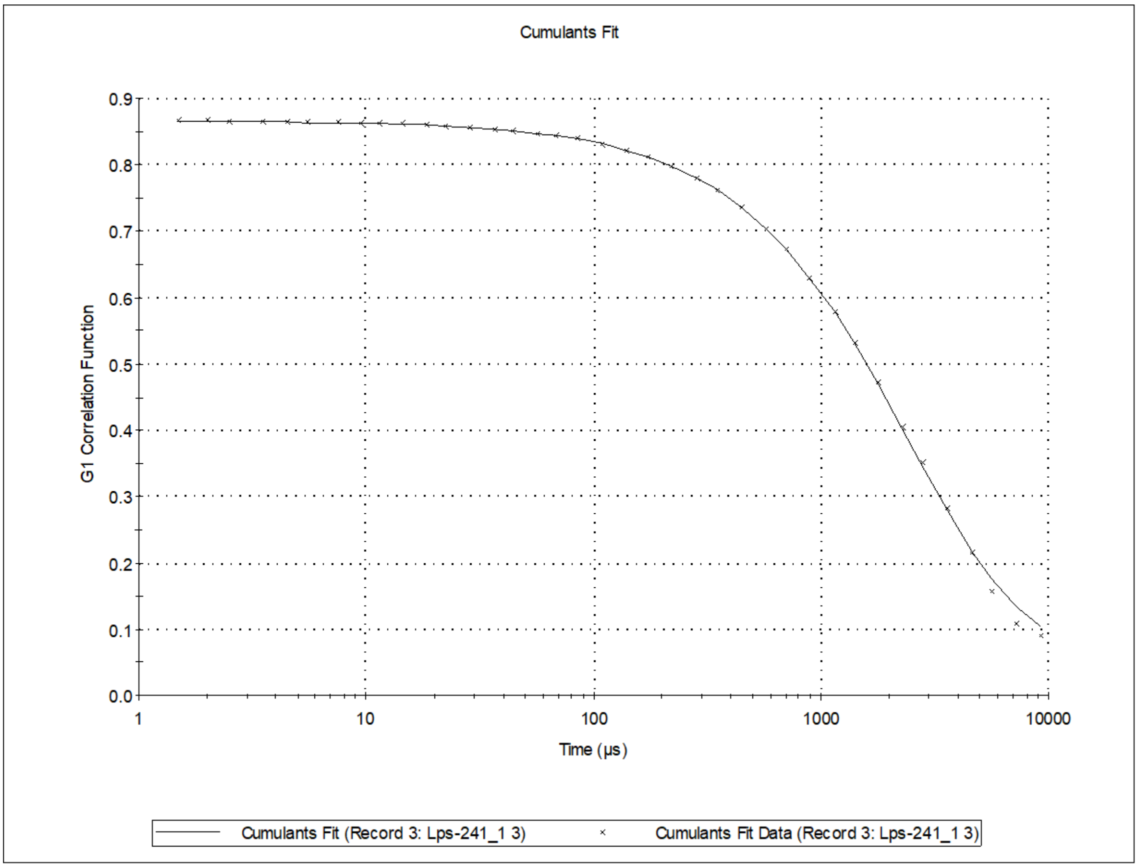

Figure S22. F-3, recrystallization from ethanol

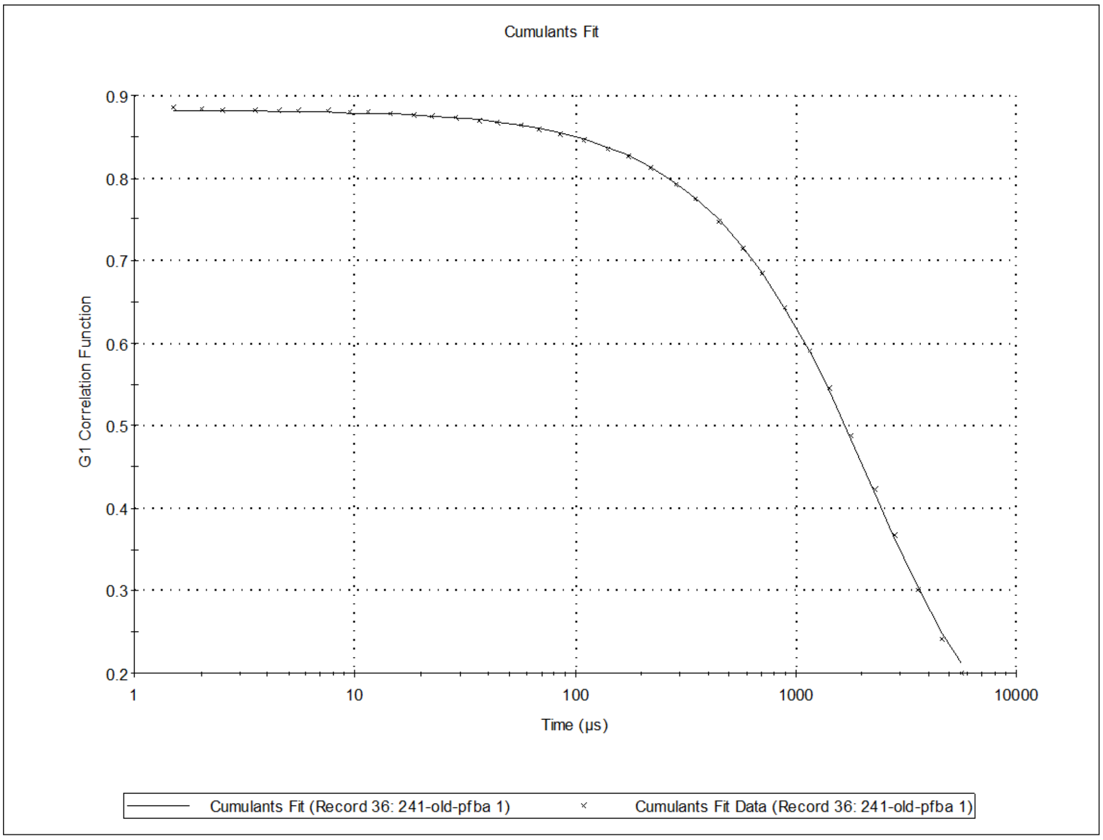

Figure S23. F-3, recrystallization from ethanol, ultrasonic

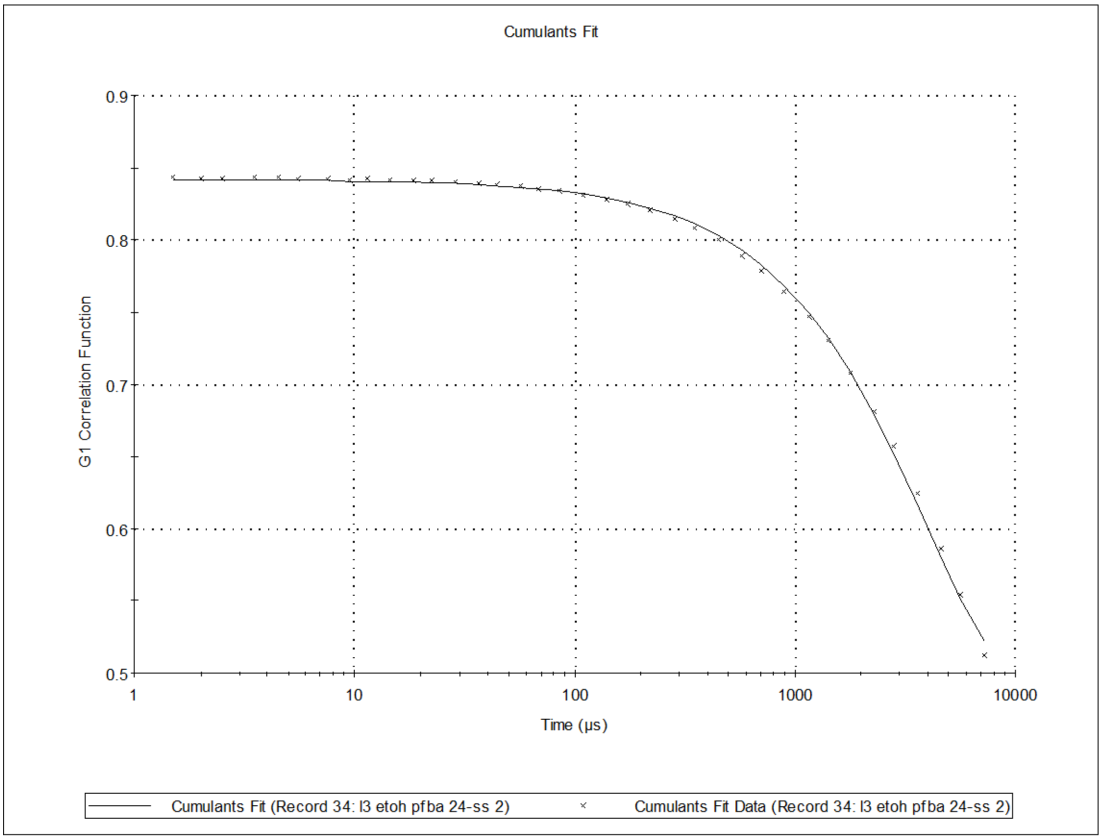

Figure S24. F-3, pentafluorobenzoic acid, recrystallization from ethanol

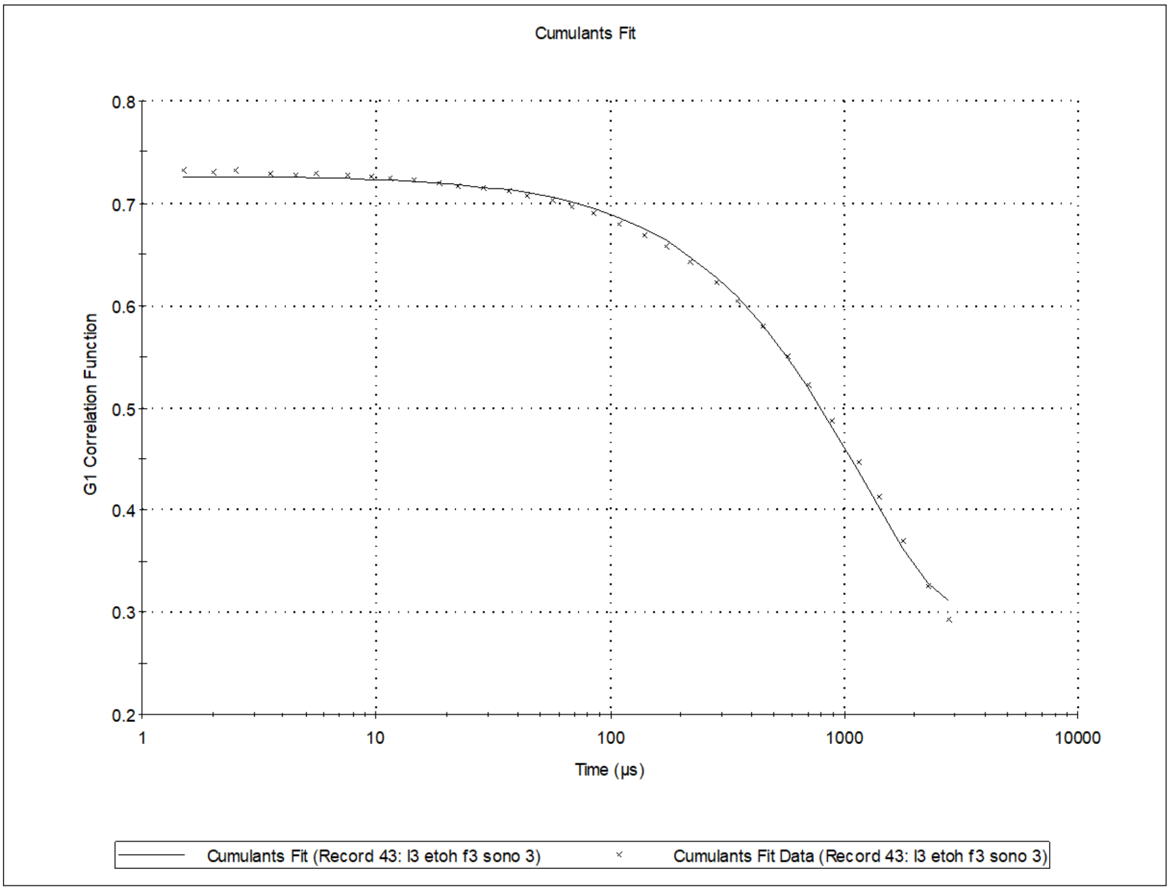

Figure S25. F-3, pentafluorobenzoic acid, recrystallization from ethanol, ultrasonic

4. Luminescence titration data

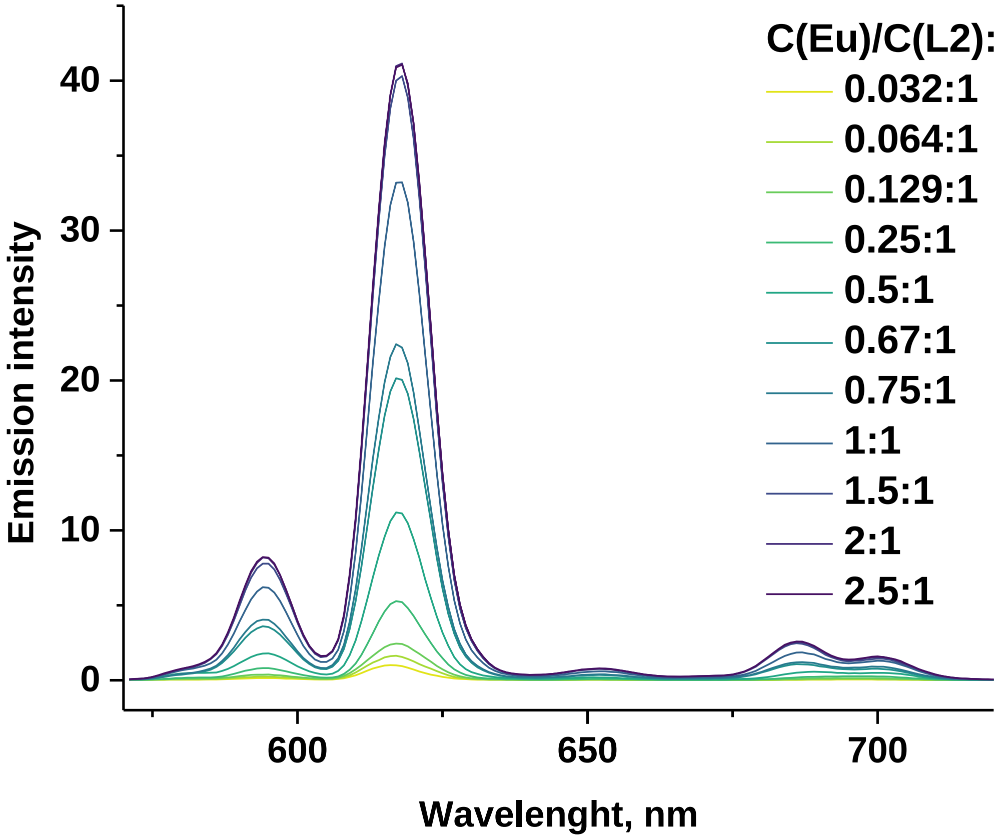

Figure S26. General view of the fluorescence spectra of europium complexes when excited at 300 nm.

5. Computation data

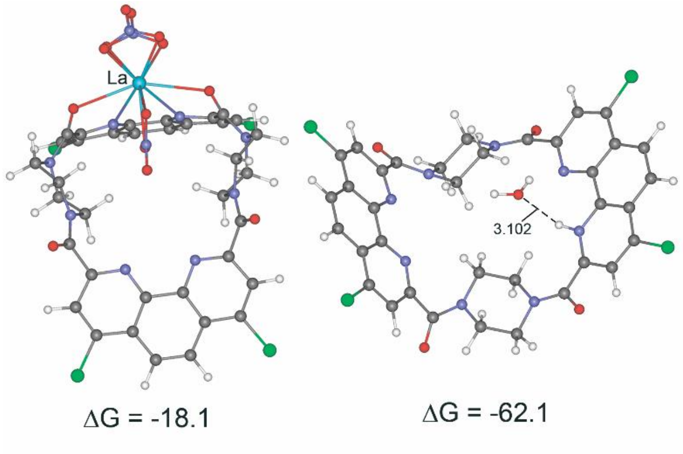

Coordinates

Conformer_1-Cl_LaNO3_AP

cartesian

set=L1

17	1.84090656	6.72923572	1.36560863
17	2.86119480	3.46323637	-5.50946429
17	-6.68276201	-2.74045058	-3.86991601
17	-7.64962789	0.39065916	2.93675765
8	-1.82727033	-4.86954266	-2.38835182
8	-2.82632664	-1.53853669	4.73600005
7	2.39577100	2.28770105	0.72221924
7	2.76756317	1.18280295	-1.60733002
7	-3.22900888	-2.22176042	-0.94943616
7	-3.57796943	-1.07408895	1.52113537
7	1.73940045	-1.66689967	-3.24760212
7	-0.63282632	-2.91336030	-2.30991599
7	0.73022274	1.59250943	3.64995946
7	-1.45019122	-0.21414062	3.46620898
6	2.02212486	5.02098584	1.08763357
6	1.81700181	4.13568272	2.13557283
1	1.53057518	4.49960797	3.12359471
6	2.00627505	2.75857252	1.89595083
6	2.50927408	3.13691945	-0.31201808
6	2.71000766	2.52499609	-1.60286964
6	2.70891349	0.54394428	-2.76562511
6	2.72803932	1.21946559	-4.00341884

1	2.71794899	0.66701661	-4.94434129
6	2.78029897	2.60586282	-3.99762939
6	2.72605464	3.32616704	-2.77255434
6	2.64461711	4.74703080	-2.62820937
1	2.70327790	5.36645227	-3.52489602
6	2.46170155	5.32959812	-1.39995228
1	2.37540623	6.41437960	-1.31602545
6	2.34148967	4.54096106	-0.21251122
6	2.67850227	-0.95884329	-2.56417352
6	1.70219855	-3.13476997	-3.11403241
1	1.47489177	-3.54373743	-4.11351425
1	2.69847575	-3.47284611	-2.79587882
6	0.64717504	-3.58837131	-2.09929749
1	0.46198368	-4.66886076	-2.19537082
1	0.99718265	-3.38228151	-1.07264265
6	-0.56612947	-1.46473998	-2.42673126
1	-0.22368721	-1.01343435	-1.47555293
1	-1.56324071	-1.07040174	-2.66688703
6	0.41800400	-1.09207903	-3.55271824
1	0.07180798	-1.49396799	-4.52053260
1	0.49255204	0.00120227	-3.62838211
6	-1.79075071	-3.64837036	-2.29665395
6	-3.09994761	-2.90136448	-2.08246125
6	-4.15007210	-3.11981737	-3.00000986
1	-3.98820254	-3.72119846	-3.89526015
6	-5.37515855	-2.55674951	-2.72017898
6	-5.58602738	-1.86214901	-1.49881659
6	-6.84907010	-1.32699137	-1.10919045
1	-7.68494626	-1.40637764	-1.80614804
6	-7.02380505	-0.76095720	0.12096020
1	-8.00144692	-0.38119441	0.42215315
6	-5.94835435	-0.68564244	1.05435505
6	-6.09924021	-0.20475404	2.38271256
6	-5.03677132	-0.22896878	3.25829371
1	-5.14885633	0.06476045	4.30253245

6	-3.78530917	-0.65483059	2.76357233
6	-4.64115809	-1.14142101	0.68942368
6	-4.45445092	-1.75092220	-0.62891421
6	-2.64416398	-0.82521782	3.75689268
6	-0.23693376	-0.64473008	4.16075921
1	0.38179887	-1.25119381	3.47624409
1	-0.55611952	-1.28341427	4.99813873
6	0.57548736	0.54760731	4.67841325
1	0.06776971	1.01514993	5.53946983
1	1.57899229	0.21460586	4.97873943
6	-0.55801697	2.02858514	3.08636074
1	-0.39164580	2.79773520	2.31989655
1	-1.17178934	2.45901836	3.89627368
6	-1.26849738	0.81571220	2.45518894
1	-2.24888902	1.12597139	2.06840288
1	-0.66387470	0.43321365	1.60993681
6	1.87472738	1.64521811	2.91694892
57	3.81621941	-0.17917218	0.64231068
8	2.80768923	0.83330821	3.00608119
8	3.49223633	-1.45229182	-1.76931966
7	5.98849649	1.75292768	-0.05184644
8	6.87294116	2.53749858	-0.33056172
8	5.27075587	1.87057053	1.02811153
8	5.68294654	0.74666971	-0.81576223
7	5.63076196	-2.16710872	1.82583746
8	6.34233465	-3.00873724	2.32883622
8	5.18382203	-1.13910750	2.49263164
8	5.25488220	-2.21977130	0.58066993
7	1.23575925	-1.71223959	1.03732391
8	0.19218640	-2.34153444	1.12160542
8	2.33316259	-2.09995936	1.57049541
8	1.28872951	-0.57424168	0.38269150

$end

Energy = -13306.7180661271

Conformer_1-Cl_H3O+

mult=1 charge=1

cartesian

set=L1

17	3.96726606	6.57708379	1.78469703
17	4.57706264	3.31773199	-5.08350526
17	-6.32761090	-3.36018909	-3.51116896
17	-7.16280790	0.08952968	3.16696675
8	-1.28311836	-4.92113017	-2.71798187
8	-2.38219475	-0.99819663	5.05156683
7	2.90873476	2.21976314	0.97134261
7	3.23258473	1.11308056	-1.44405299
7	-2.79413124	-2.47411458	-0.79068061
7	-3.10089744	-1.27915636	1.64588593
7	2.07544945	-1.67105455	-2.83173876
7	-0.17511280	-3.09586061	-1.89605059
7	1.24784234	1.23910663	3.42488412
7	-0.82120771	-0.69668924	3.40938066
6	3.58327705	4.91958725	1.45665488
6	3.14574073	4.09697804	2.47259712
1	3.07316180	4.45800852	3.49906050
6	2.79414644	2.75459681	2.18442783
6	3.38305257	3.02247746	-0.00942166
6	3.53768707	2.43752760	-1.30949327
6	3.34303725	0.44058433	-2.60284577
6	3.76748141	1.13700360	-3.74181316
1	3.87730319	0.59867210	-4.68328170
6	4.07123638	2.48786929	-3.66041090
6	3.98026630	3.18824424	-2.42104622
6	4.30346985	4.56219592	-2.22542511
1	4.65405735	5.14503365	-3.07785988
6	4.18632964	5.13677255	-0.98675815
1	4.44740653	6.18678518	-0.84264259
6	3.72762931	4.39340679	0.14049200
6	3.27334600	-1.07752890	-2.63688831

6	2.01408678	-3.12885729	-3.03316210
1	1.60965399	-3.31871244	-4.04249599
1	3.04093509	-3.51660880	-2.97219390
6	1.11321413	-3.77728077	-1.97977398
1	0.90869706	-4.82278164	-2.25432938
1	1.60744212	-3.74488182	-0.99171552
6	-0.08702888	-1.66358395	-1.67039878
1	0.36342449	-1.46706503	-0.67868205
1	-1.09385372	-1.23087917	-1.66801656
6	0.77412343	-1.00310871	-2.76167216
1	0.27003300	-1.10004212	-3.73998694
1	0.89349183	0.06584869	-2.52512334
6	-1.31839717	-3.79701636	-2.23449377
6	-2.66374863	-3.17134344	-1.91339597
6	-3.73425392	-3.49818801	-2.77106736
1	-3.55776301	-4.11469601	-3.65265010
6	-4.98922858	-3.02999176	-2.44484860
6	-5.19303329	-2.30849225	-1.23665401
6	-6.46977944	-1.84404465	-0.80330954
1	-7.33300701	-2.01491577	-1.44849198
6	-6.61965642	-1.22304863	0.40409028
1	-7.60433891	-0.88980166	0.73591077
6	-5.50688623	-1.02529789	1.27402787
6	-5.61916002	-0.46297342	2.57483467
6	-4.51246989	-0.37925698	3.39081835
1	-4.57492716	-0.02716165	4.42035782
6	-3.26749227	-0.79536084	2.87138966
6	-4.19707891	-1.42978360	0.86886693
6	-4.03501204	-2.08159904	-0.42868253
6	-2.11744622	-0.81324309	3.86842887
6	0.26344675	-0.81022188	4.38517900
1	1.03204233	-1.49358865	3.98373091
1	-0.16438156	-1.23064327	5.30614599
6	0.87306615	0.56072473	4.67643014
1	0.13588308	1.18294088	5.21623651

1	1.78195641	0.47670367	5.28843477
6	0.18170356	1.32284181	2.42358856
1	0.57242758	1.73402334	1.48274048
1	-0.62301972	1.98330725	2.80086220
6	-0.40579746	-0.07438570	2.16004151
1	-1.26431508	-0.02493010	1.47944192
1	0.36606363	-0.71037489	1.68677566
6	2.43637572	1.90368748	3.39047929
8	3.26553446	1.90417021	4.29696252
8	4.36844480	-1.62586705	-2.56425776
8	0.04252513	1.77045963	-1.06306493
1	-0.11837480	2.67656614	-1.38381269
1	-0.85885059	1.43765847	-0.89860441
1	2.90424165	0.68297704	-0.55978084

$end

Energy = -4047.1934026583
